# Applications of Hybrid Polymers Generated from Living Anionic Ring Opening Polymerization

**DOI:** 10.3390/molecules26092755

**Published:** 2021-05-07

**Authors:** Jonathan Goff, Santy Sulaiman, Barry Arkles

**Affiliations:** Gelest Inc., 11 Steel Road East, Morrisville, PA 19067, USA; ssulaiman@gelest.com (S.S.); executiveoffice@gelest.com (B.A.)

**Keywords:** hybrid polymers, ring-opening polymerization, contact lenses, breathable films, membranes, high elongation elastomers, biomimetic polymers, photoresists

## Abstract

Increasingly precise control of polymer architectures generated by “Living” Anionic Ring-Opening Polymerization (Living AROP) is leading to a broad range of commercial advanced material applications, particularly in the area of siloxane macromers. While academic reports on such materials remain sparse, a significant portion of the global population interacts with them on a daily basis—in applications including medical devices, microelectronics, food packaging, synthetic leather, release coatings, and pigment dispersions. The primary driver of this increased utilization of siloxane macromers is their ability to incorporate the properties of silicones into organic structures in a balanced manner. Compared to organic polymers, the differentiating properties of silicones—low *T_g_*, hydrophobicity, low surface energy, and high free molal space—logically lend themselves to applications in which low modulus, release, permeability to oxygen and moisture, and tactile interaction are desired. However, their mechanical, structural and processing properties have until recently precluded practical applications. This review presents applications of “Living” AROP derived polymers from the perspective of historical technology development. Applications in which products are produced on a commercial scale—defined as not only offered for sale, but sold on a recurrent basis—are emphasized. Hybrid polymers with intriguing nanoscale morphology and potential applications in photoresist, microcontact printing, biomimetic soft materials, and liquid crystals are also discussed. Previously unreported work by the authors is provided in the context of this review.

## 1. Introduction

**Figure 1:**
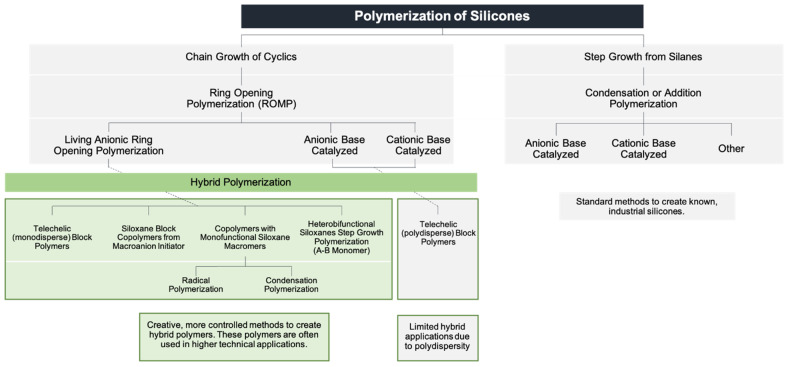
Synthetic methods for preparation of siloxane and siloxane-hybrid polymers.

Functional siloxane polymers constitute a large class of reactive materials. Siloxanes with vinyl, silanol, and hydride substitution are the most widely utilized, serving as the basis for the majority of elastomeric silicone products. Functional siloxanes combined with organic monomers form “hybrid” polymers which, despite their demonstrated utility, have comparatively limited commercial applications. Most siloxane polymers are prepared by ring-opening polymerization with high degrees of polydispersion, thereby curtailing their ability to act as precise structural elements. On the other hand, the economics of both the basic building blocks and the polymerization process itself favor equilibrium-derived siloxane polymers. Figure 1 depicts the range of synthetic methods utilized to prepare siloxane polymers.

Due to the intrinsic process as well as the structural control that it provides, Living AROP-derived polymers provide the potential for a broad range of hybrid organic–inorganic materials. Briefly, AROP-derived materials provide a mechanism for translating macromolecular synthetic methods normally associated with organic polymers (and excluded from inorganic polymers) into hybrid polymer structures. Among these AROP-derived siloxane polymers, perhaps the most technologically significant are monofunctional and heterobifunctional silicone macromers. Siloxane macromers are defined as silicon-containing species with a single functional polymerizable group which, although used as monomers, possess sufficiently high molecular weight and enough internal monomer units to be considered polymeric. In another sense, they are siloxane building blocks derived from Living AROP. Siloxane macromers enable the use of technologies other than those associated with siloxane polymerization—e.g., techniques associated with the wider range of synthetic organic polymerization technologies—to incorporate siloxane-associated properties: e.g., techniques associated with the wider range of synthetic organic polymerization technologies. Notably, only controlled “living” AROP provides a path to siloxane polymers with sufficiently controlled structures and functionality to behave as macromers in polymerization with organic monomers. Siloxane macromers thus enable the introduction of selected siloxane properties into higher order structures via macromolecular engineering.

Although the advent of living anionic ring-opening polymerization can be traced back approximately 50 years, commercial applications of this technology have only reached the marketplace within the last 20 and, by our estimate, production has exceeded 100 tons per annum only in the last 5. The availability and range of polymers with tailored molecular weight, polymer backbone structure and basic architecture, and both functional and non-functional alternatives have grown enormously, stimulated by symbiotic application and development efforts. This review relies heavily on patent literature; the global collection of patents, of which there are over 10,000,000 in the US alone, is one of the most comprehensive collections of technical information in the world but is often neglected in scholarly reviews. Nevertheless, the impact of patent technology on how materials are prepared and utilized may equal or exceed that of academic literature.

### 1.1. Fundamentals/Building Blocks/Architectures

**Scheme 1:**
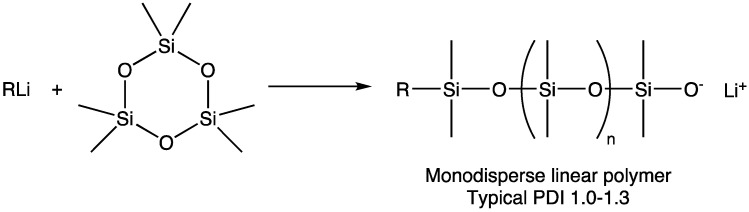
Kinetically Controlled Polymerization.

**Scheme 2:**
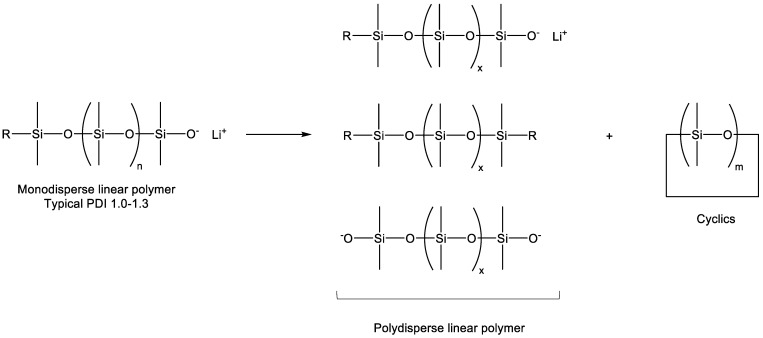
Thermodynamic Equilibrium Driven Processes (i.e., Reversion, Backbiting, Chain-scrambling).

The “living” anionic ring-opening polymerization of cyclosiloxanes is in fact more properly described as kinetically controlled ring-opening polymerization. The features that define the “living” aspects of the polymerization are: a quantitative initiation (as shown in Scheme 1), and the fact that the rate of polymerization propagation is significantly greater than that of the polymer chain randomization processes, particularly the reversion of the degree of polymerization driven by equilibration or “back-biting” processes (as shown in Scheme 2) [1].

Both initiation and back-biting are driven by the catalytic opening of a siloxane bond. The evolution of Living AROP has depended on the recognition of classes of cyclic siloxane monomers that possess ring strain, as well as on “weak” catalysts which are able to rapidly cleave the Si-O-Si bonds of the strained monomers but are relatively slow and ineffective at cleaving Si-O-Si bonds in unstrained systems. The difference in reaction kinetics provides an opportunity to deactivate the catalyst before significant equilibration effects are observed, resulting in the scalable preparation of polymers with polydispersities approaching 1.

**Scheme 3:**
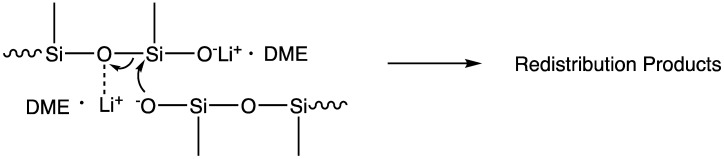
Ion-pair Complexation by Dimethoxyethane (DME) Promoter Favors Chain-End Over Internal Scrambling [5].

The differential polymerization of ring-strained cyclics, as opposed to unstrained cyclic siloxanes, was apparently observed in early industrial development, as is made particularly clear in the example of fluorinated silicones generated from 3,3,3-trifluoropropylmethylcyclic siloxanes [2]. Attempts to polymerize cyclic tetrasiloxanes were ineffective due to the fact that the reversion kinetics apparently matched those of polymerization for the unstrained cyclic tetramer. The polymerization of cyclic trisiloxanes, on the other hand, was effective due to the ability to deactivate the catalyst before significant reversion could occur. The potential for weak catalysts such as lithium phenoxide to produce polymers of ring-strained monomers was first recognized by McVannel [3], while the quantitative, selective formation of a lithium initiator generated from the reaction of organolithium reagents with cyclic trimers was studied by Frye [4], who, surprisingly, observed that, in a 1:1 molar stoichiometry, *n*-butyl lithium reacted with hexamethylcyclotrisiloxane (D_3_) to form lithium n-butyldimethylsilanolate, leaving 2/3 of the D_3_ unreacted. Lee and Frye also noted that polar “promoters” would then cause polymerization to proceed [5]. Finally, Fessler reported the relative effectiveness of promoters in the living polymerization process and provided mechanistic insight into siloxane-silanolate reactions that could result in shifts between triad, Gaussian, and redistribution products, as shown in Scheme 3 [6].

**Figure 2:**
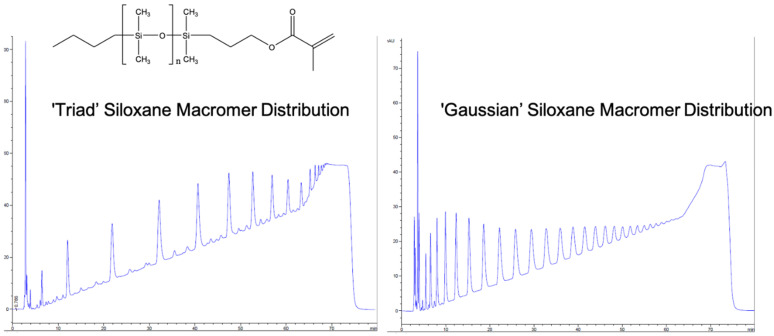
Comparison of silicone macromers demonstrating polymer termini scrambling (GC of methacrylate functional macromer (MCR-M11) with a nominal MW of 1000 showing shift from Triad to Gaussian distribution with a change in promoter. (Author’s work)).

A comparison of a GC (gas chromatograph) of siloxane macromers with equivalent MW and PDIs, showing triad and Gaussian distributions, is provided in Figure 2 (author’s work). In contrast to anionic polymerization with K^+^ and Na^+^, in which there is little differentiation between chain scission points, the Li^+^ redistribution mechanism favors chain termini: i.e., despite chain-end scrambling, narrow polydispersity is maintained.

**Figure 3:**
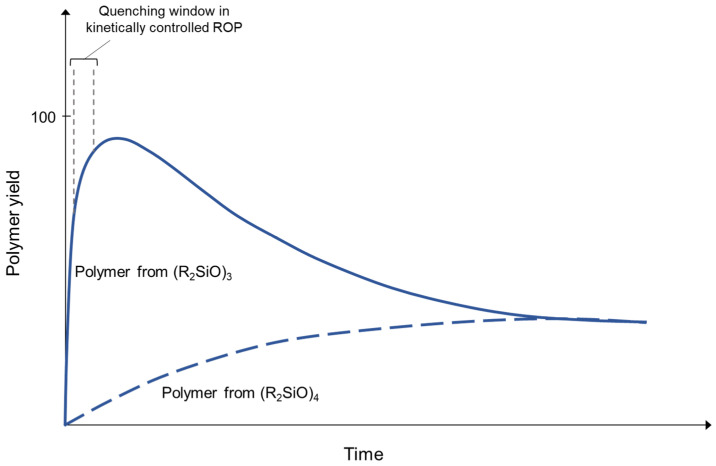
Time-dependent ROP polymer growth of strained versus unstrained cyclosiloxanes.

During the same period when Lee and Frye’s report was published but separate from the discoveries relating to ring-strained siloxane monomers and lithium-based initiators, there was significant interest within the silicone industry in generating block copolymers, with lithium silanolate-based initiators being shown to lead to the sequential polymerization of cyclotrisiloxanes. The combination of the growing interest in forming block copolymers, the development of siloxanes with strained cyclic structures, and quantitative lithium catalyzed polymerization underlies the publication by Saam [7,8] that reviewers point to as establishing the potential of “living” AROP siloxanes—in which he clearly demonstrated initiation, promotion, narrow MW distribution, and the ability to form block copolymers. It was recognized at that time that these polymers had to be terminated before equilibrium processes dominated in order to maintain the target molecular weight, as visualized in Figure 3. Functionalized termination reagents were later used to create siloxane macromers [9,10], although a process for preparing these macromers was not reported until 10 years later [11].

**Scheme 4:**
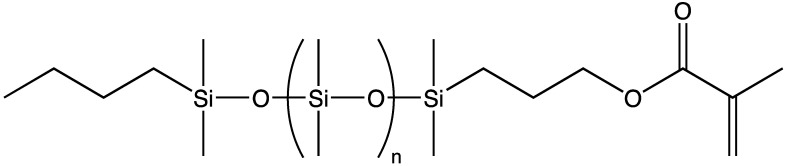
Polydimethylsiloxane macromers with symmetric architectures, produced via the anionic ring-opening polymerization of a cyclic trisiloxane and subsequent coupling with a functional dihalogen-substituted silane reagent, have recently been described.

**Scheme 5:**
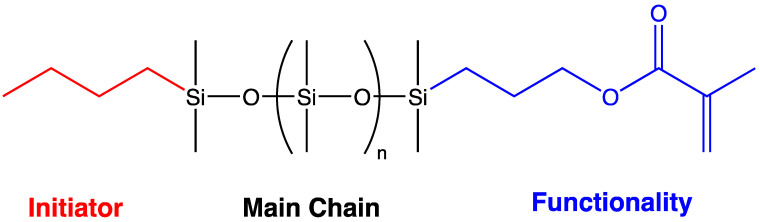
Siloxane Macromer Structural Segments.

**Scheme 6:**
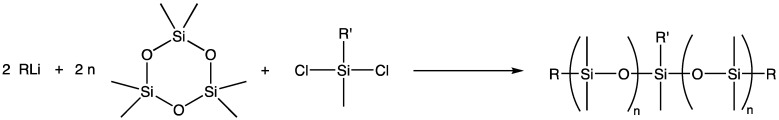
Symmetric macromers can contain a pendant methacrylate group bisecting the polydimethylsiloxane backbone.

**Scheme 7:**
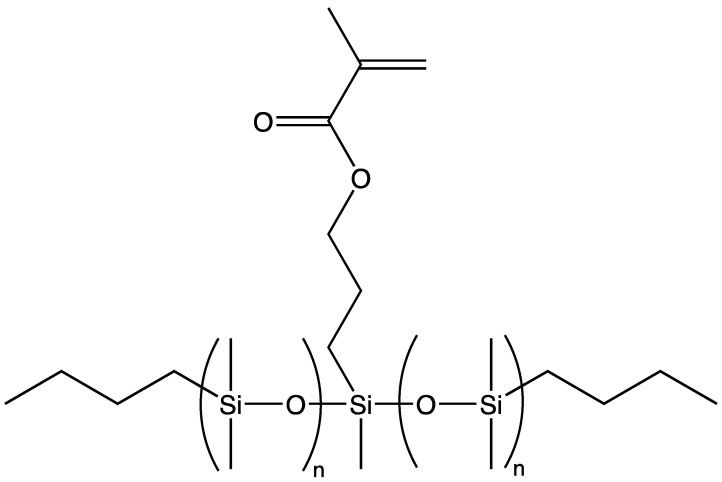
Symmetric silicone macromer with methacrylate functionality bisection.

**Scheme 8:**
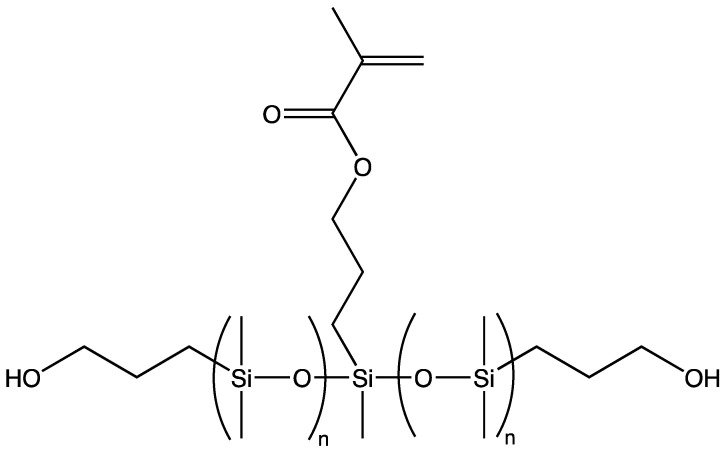
Carbinol terminated telelechelic with macromeric methacrylate functionality.

The term “macromer” is a contraction of the word macromonomer and refers to a relatively high molecular weight species with a single functional group which, although used as a monomer, has sufficient internal monomer units to be considered a polymer. The earliest commercial siloxane macromers contained methacrylate functionality and found commercial utility in the formation of organic–inorganic hybrid polymers (Scheme 4). Their termination, or “capping”, functionality was derived from the use of methacryloxypropyldimethylchlorosilane. The general structure for a siloxane is depicted in Scheme 5. Variations of the basic structure are depicted in Scheme 6, Scheme 7 and Scheme 8.

A second class of functionality can be introduced into macromers by using novel initiators, thereby yielding telechelic polymers in which the second functional class—e.g., hydroxyl—is located at the telechelic polymer termini, which are equidistant from the first functional class.

More recently, living AROP has been combined with the concept of functional initiators to generate both monodisperse telechelic and heterobifunctional siloxanes.

This historic overview has only given a condensed description of the chemistry, structure, and function of siloxanes derived via living AROP. For those interested in more details regarding the chemical aspects of siloxanes derived from living AROP, the following references should be consulted [1,12,13]. While the bulk of the literature and commercial applications utilize a lithium anion as a weak base component of the initiator, an intriguing recent series of reports utilize substituted cyclic guanidines in combination with water or silanol that act as initiators for living AROP [14,15].

The following tables summarize monomeric building blocks (Table 1), the initiators (Table 2), functional terminations, and architectures that have shown practical utility in commercial applications.

### 1.2. Monomeric Building Blocks

The fact that there are only five monomeric building blocks for the living AROP-derived polymers that represent virtually all commercial applications as well as the vast majority of published reports is a consequence both of practical synthetic routes [16,17,18] and the sluggish rates of polymerization reported for cyclotrisiloxanes with greater organic substitution [19]. A preparation has been reported [20] for the simple and highly desirable monomer trimethylcyclotrisiloxane, but its practical isolation has not yet been described. This has led to interest in pentamethylcyclotrisiloxane [21] and hydridotetramethylsiloxanylethyl-substituted cyclotrisiloxanes [22], which can be used directly as monomers for polymerization or reacted with various olefins to form more elaborately substituted trisiloxanes. Similarly, vinylpentamethylcyclotrisiloxane has been prepared, and offers an advantage over trivinyltrimethylcyclotrisiloxane in cases where isolated vinyl substitution on the polymer chain is desired [23]. The primary cyclic monomers used in the production of macromers on both commercial and research levels are listed for convenience in Table 1. Other cyclic monomers have been reported in the literature, and include higher hexaalkylcyclotrisiloxanes [24], chloropropylmethylcyclotrisiloxanes [25], and substituted hexaarylcyclotrisiloxanes [26]. Specialty monomers with limited reference include D_4_ [27], acrylate [28], cyclic ether [29], trimethylsiloxy-substituted [30] cyclotrisiloxanes, and similarly substituted strained carbosiloxanes [31].

### 1.3. Termination

Terminating molecules in living AROP can serve several functions. At a minimum, they serve to quench the polymerization before redistribution processes associated with reversion, interchain back-biting, and interchain scrambling; their potential functions can be further divided into the following categories:

Non-functional termination;
Non-functional coupling to effect non-functional doubling of MW;
Non-functional coupling with block insertion;
Functional termination to form asymmetric macromers;
Functional coupling to form symmetric macromers;
Functional coupling to form branched structures.

Terminators in AROP processes are usually chlorosilanes, although other halosilanes and alkoxysilanes have been reported. Apart from the terminators found in the literature associated with living AROP of siloxanes, terminators normally associated with the living anionic polymerization of olefins and other organic polymers can provide similar termination for siloxanes [77,78].

### 1.4. Macromers Commonly Reported in Literature

The structures and properties listed in Table 3 are an aggregate of the most common siloxane macromers reported in the scholarly and commercial literature. They should not be considered exact values, but rather nominal values for similar materials. Similarly, heterobifunctional macromers are reported in Table 4.

## 2. Applications

### 2.1. Compatibility and Reactivity Introduction

The living polymerization of siloxanes provides the basis for synthesizing siloxane macromers capable of acting as precise structural elements, achieving both the requirements of copolymerization with organic monomers as well as the desired properties of copolymers. The incorporation of properties associated with siloxanes—including oxygen and moisture permeability, release, or thermal performance—would thereby improve the performance of a wide range of organic polymers. To date, however, the utilization of silicone macromers in combination with organic monomers has been primarily limited by economic considerations, as well as by structural and compositional challenges.

The most commonly used siloxane macromers are methacrylate-terminated, both in the literature and in commercial applications. Reported reactivity ratios [79] and previously unreported solubility in monomers are shown in Table 5 and Table 6, respectively

### 2.2. Gas/Vapor Permeability

In comparison to organic polymers, siloxanes possess a large free molal volume, a consequence of the length of both silicon–oxygen and silicon–carbon bonds, which allows greater permeation of small molecules. These same factors contribute to the flexibility of silicones which imposes limitations on structural properties. Hybrid polymer systems utilizing both macromer and block polymers generated by living AROP provide the balance of properties required in contact lenses, breathable films, and membranes.

#### 2.2.1. Contact Lens/Hydrogel

**Figure 4:**
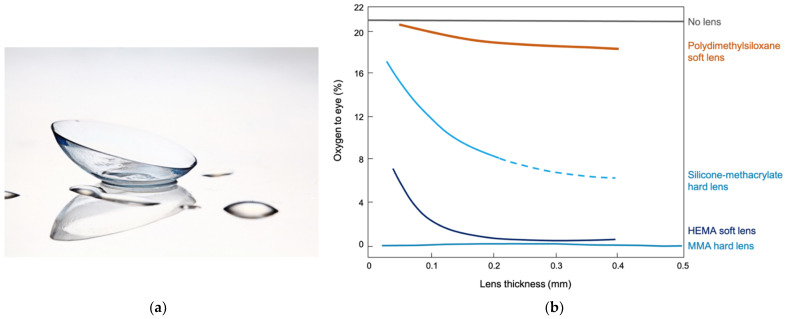

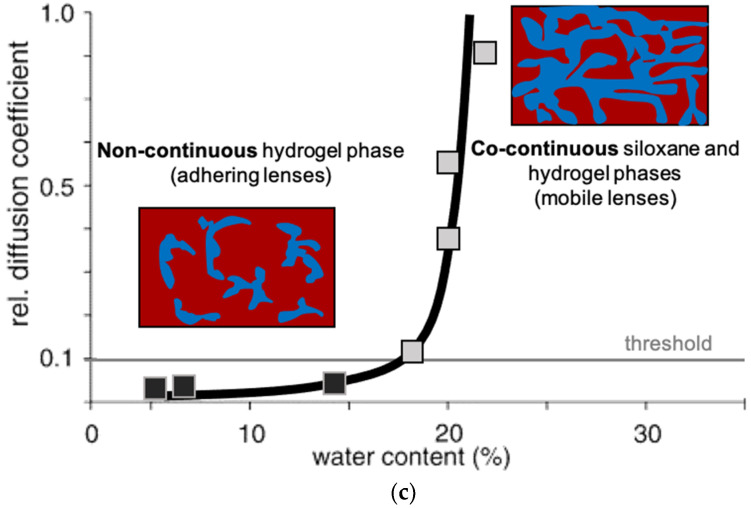
(**a**) Silicone hydrogel lenses incorporate low MW macromers into otherwise hydrophilic matrices based on hydroxyethylmethacrylate or dimethylacrylamide; (**b**) comparison of contact lens materials and oxygen transport to the cornea (redrawn with permission from Ref. [122] Copyright 1983 the Technomic Publication). (**c**) Hydrogel phase structure as a determinant of lens comfort (redrawn with permission from Ref. [121] Copyright 2001 Elsevier Science).

Oxygen permeability is a key feature of modern contact lenses, particularly those intended for extended wear. In addition to the bloodstream, the corneal and scleral tissues of the eye take about 1/3 of their respiratory requirement directly from the air. Methacrylate-derived polymers, while possessing good optical and mechanical properties, are occlusive to oxygen transport due to the fact that the permeability of siloxanes is more than 100X greater than that of analogous hydrocarbon structures. Gaylord’s pioneering material work [117,118] under the direction of Seidner led to the commercialization of rigid gas permeable contact lenses by Syntex in 1980 [119]. While these lenses satisfied the physiological requirement for extended wear, broad acceptance of silicone rigid gas permeable (RGP) lenses was not achieved, since user comfort did not match that of hydrogel lenses. Formulation and process challenges in silicone hydrogel contact lens manufacture include combining hydrophobic silicones with hydrophilic monomers while maintaining optical clarity, high water content, and high oxygen transport to the eye in the resulting hydrogels. The first successful silicone hydrogel lens, based on technology disclosed by Vanderlaan, was introduced to the marketplace by Vistakon in 2004 [102]. Monofunctional siloxane macromers polymerized by living anionic polymerization routes are key materials in such silicone hydrogel contact lens formulations: these monodisperse monofunctional materials are used as comonomers with hydrophilic hydrogel monomers such as hydroxyethylmethacrylate (HEMA) and dimethylacrylamide (DMA). The earliest report of soft silicone hydrogel lenses generated from siloxane macromers and HEMA that displayed acceptable performance utilized group transfer polymerization [120]. One material requirement of a silioxane macromer selected for such a formulation is a minimal solubility in the hydrophilic hydrogel monomers. It has been reported that, after curing the silicone hydrogel contact lens reactive monomer mix, an optically clear co-continuous silicone and hydrogel phase membrane suitable for contact lens use is formed [121]. Incorporating low molecular weight (~1000 g mol^−1^) siloxane macromers into silicone hydrogel formulations enhances oxygen permeability in the resulting contact lenses, achieving superior oxygen transport to the eye compared to hard rigid gas permeable (RGP) and soft HEMA contact lens technologies (Figure 4) [122].

During the contact lens molding process, extraction procedures are employed to remove undesirable impurities. Water insoluble impurities, for example, decrease optical clarity and leach out of the contact lens, causing negative ocular effects such as stinging [92]. Unfortunately, the hydrophobic nature of these impurities makes conventional water extraction procedures insufficient. Silicone hydrogel extraction procedures using alcohols have been reported, but possess drawbacks including increased manufacturing costs, organic solvent waste handling concerns, and potential eye irritation from residual solvent. Living anionic polymerization enables the synthesis of monodisperse monofunctional siloxane macromers that are largely free of impurities. Indeed, newer generation silicone hydrogel formulations employ siloxane macromers of sufficient purity to rely solely on aqueous extraction and hydration steps post-molding. Table 7 gives an overview of the different generations of silicone hydrogel technologies. Generation 1 silicone hydrogel contact lenses did not use siloxane macromers and relied on tris(trimethylsilyl)-silylpropylmethacrylate (TRIS) and difunctional silicone crosslinkers as the oxygen transport enhancers in the formulation. TRIS made control of the lenses’ mechanical properties difficult, resulting in higher modulus lenses that were not comfortable for the wearer [121]. Generation 2 and 3 silicone hydrogel formulations incorporated siloxane macromers as oxygen transport enhancers, which led to greater formulation flexibility and control of the final contact lens material properties (e.g., Dk and modulus) [100,101,123].

Hydrogels comprised of macromers with both polycarbosiloxane units and poly(trifluoropropylmethyl)siloxane units have been reported. The macromers are synthesized by substituting D_3_ with other ring-strained monomers in a living polymerization process. Awasti describes a monomethacrylate functional polycarbosiloxane synthesized from a 2,2,5,5-tetramethyl-2,5-disila-1-oxacyclopentane monomer as resistant to hydrolysis and therefore suitable for use in silicone hydrogel soft contact lens design [55,128]. Monomethacrylate functional poly(trifluoropropylmethyl)siloxane macromers possess increased polarity, improving their miscibility with hydrophilic monomers and potentially reducing the non-specific binding of proteins on soft contact lens surfaces [59,70].

Low molecular weight symmetric siloxane macromers with hydrophilic termini have increased miscibility with hydrophilic monomers compared to asymmetric siloxane macromers, as the smaller siloxane block size of the symmetric architecture limits phase separated domain formation while still maintaining the oxygen transport-enhancing benefits of asymmetric analogs. The hydrophilic termini reported by Kimble include methoxypropyl and hydroxypropyl groups [59,60]. α-Methacrylate functional, ω-polyalkyleneoxide siloxane macromers have also been described; however, their block copolymer structure results in microphase separation, rendering this macromer structure unsuitable for the production of optically clear hydrogel films [129].

One approach for improving contact lens comfort is to utilize a plasma treatment to improve hydrogel wettability and then apply hydrophilic terpolymers derived from combinations of silicone macromers and hydrophilic monomers such as diethylazetidinium methacrylate ester chloride salt, thereby providing permanent wettability [130].

In a separate but related area, siloxane-based interocular lenses (IOL) provide new opportunities for macromers. Hydride terminated macromers are utilized to control the modulus of cured elastomers used in IOLs [131]. Interestingly, silicone macromers are broadly described as components in laser adjustable IOLs that enable a post-insertion change in dioptric power by altering the refraction of the lens material [132].

Independent of contact lens development, optically clear films were derived from copolymers of styrene, ethylene glycol dimethacrylate (EGDMA), and siloxane-urethane-methacrylate (SiUMA). The SiUMA monomer was synthesized from carbinol-terminated siloxane macromers reacted with isocyanatoethylmethacrylate [133].

#### 2.2.2. Controlled Atmospheric Packaging—Modified Atmosphere Packaging

**Figure 5:**
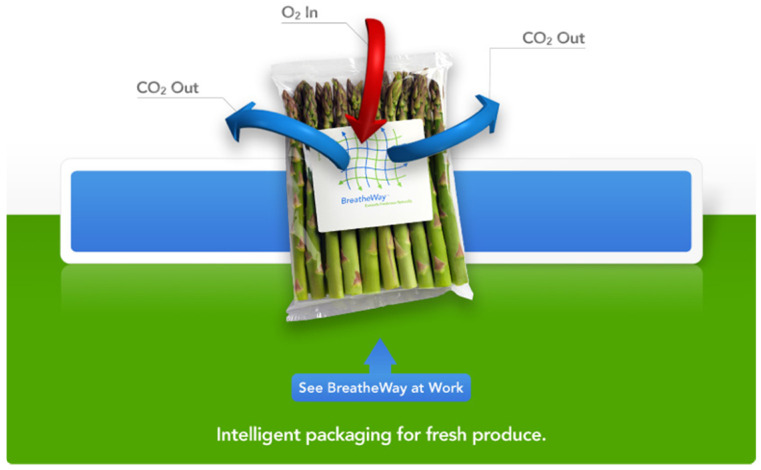
Breathable films and packaging (Reprinted courtesy of Breathway Corp).

An appreciation of the role that gases play in maintaining the freshness of meats, fruits, and vegetables during transport has created a role for polymer films with controlled permeability. Optimal oxygen concentration is associated with the appearance of “red” meat and “green” vegetables. In the case of meat, deoxymyoglobin, which is purple, forms when metabolic, diffusion, and other processes deplete oxygen availability. Over time, however, oxymyoglobin is oxidized to amber-brown methemoglobin, which is associated with a lack of freshness [134]. Ethylene is associated with fruit ripening and abscission. Gas permeable films derived from acrylate terpolymers act as overlayers to porous structures, maintaining the oxygen transmission of these structures while providing a barrier to microbial infiltration. Unlike in the case of contact lenses, transparency is usually not required for packaging applications. The use of higher molecular weight monomethacrylate macromers in packaging is therefore acceptable, and has been reported [135]. Breathable films based on this technology have become components in commercial packaging applications offered by BreatheWay, as shown in Figure 5.

### 2.3. Membranes

The utilization of polydimethylsiloxanes and silicon-rich homopolymers such as poly(trimethylsilylpropyne) [136] and poly(vinyltrimethylsilane) [137] for gas separation membranes has been established. The removal of volatile organic compounds from aqueous mixtures has been more effectively addressed by hybrid polymer systems in which there is formation of siloxane microphases in continuous organic phases. Reports on graft polymer and interpenetrating polymer networks were able to demonstrate selective pervaporation and removal of organics from aqueous streams [138,139]. The control of selectivity and permeation rates has been accomplished with copolymers derived from methacrylate functional macromers copolymerized with a variety of other methacrylate monomers. Uragami demonstrated that macromers with molecular weights of ~4000 Daltons formed copolymers with methyl methacrylate which, depending on comonomer content, allowed selective permeation of water or ethanol from ethanol–water solutions by varying comonomer contents [140]. In a series of reports, Urgami extended these systems to styrene copolymers and the incorporation of ionic liquids, enabling the selective pervaporation of volatile organics, including toluene and chlorinated organics, from aqueous streams [141,142,143].

### 2.4. Surface Properties/Modification

#### 2.4.1. Dyes, Micelles and Particles for Advanced Printing, Reprographics, and Lithography

Methacrylate functional macromers have been used in both organic dye [89] and pigment-based printing applications [98,99,144]. While these technologies are quite different, the role of the silicone macromer has common features in each. In ink-jet applications, a bulk solvent-based polymerization with dye is accomplished, after which micelles are formed via the addition of water and simultaneous evaporation of solvent. In pigment-based applications, the polymer forms an encapsulant or binder. In both cases, the silicone macromer appears to serve as a hydrophobic outer surface of the micelle or pigment, thereby helping to control particle charging and, secondarily, contributing to the spread and adhesion of the micelles or particles on the substrate. Polymersomes with controlled architecture in both their overall dimensions and membrane thickness have been prepared from diblock polymers of cabinol-termined siloxane macromers and methyloxazolidine [82].

Interestingly, while the ability of styrene-PDMS deblock polymers generated via living AROP to form micelles was recognized during early development efforts [145], no recent commercial applications have been reported.

**Figure 6:**
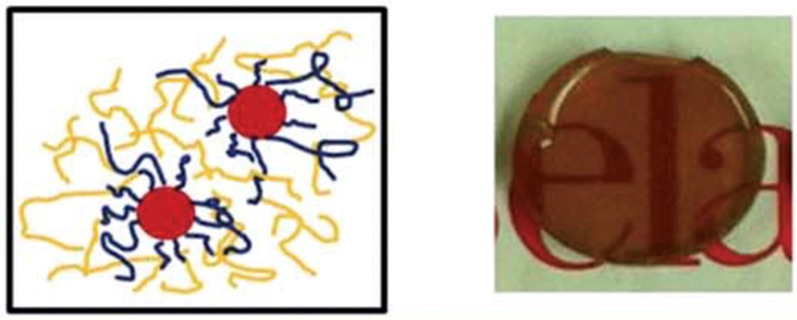
Electroluminescent transparent quantum dot composites (Tao) [85]. Left: depiction of macromers (blue) modifying a CdSe QD (red) in a silicone matrix; Right: specimen of composite. (Reprinted with permission from Ref. [85] Copyright 2013 the Royal Society of Chemistry).

Colored fluids for electrowetting and electrofluidic applications have also been generated from highly polar dyes by reaction with aminopropyl-terminated siloxane macromers [80]. For example, the yellow dye 2-(4-carboxyphenylazo)acetoacetanalide reacted with a 1000 Mn amino-terminated macromer to form the product 4-(E)-(2,4-dioxopentan-3-yl)diazenyl-N-polydimethylsiloxane-benzamide, which was soluble in non-polar fluids including silicones. Radiation-curable films for adhesive and lithographic applications, in which aminopropyl-terminated siloxane macromers are acrylated, were reported by Leir [146]. Carbinol-terminated siloxane macromers were converted to phosphate-terminated macromers by Tao and then reacted with the surfaces of CdSe quantum dots before incorporation into bulk silicones to form electroluminescent transparent films, as shown in Figure 6 [85].

#### 2.4.2. Coatings Additives—Leveling Agents, Clean Surfaces and Release

Siloxane macromer block polymers are offered commercially as leveling agents to reduce waviness and orange peel in organic coatings. The effect occurs at low concentrations and is based on activity at the liquid–gas interface, in which these polymers are oriented due to limited incompatibility with the actual binder component of the coating system [147,148]. Their versatile chemistry and modular molecular structure make it possible to adjust the properties of these macromers for specific applications. For example, further improvements in levelling have been reported when a fluorinated oxetane is reacted with unsaturated termination in macromers [149].

High silicone content macromers can impart anti-graffiti properties to coated surfaces, though this often requires concentrations higher than those used in levelling applications. Macromers with a lower silicone content can reduce surface tension and improve substrate wetting without impairing recoatability. In the case of automotive coatings, other benefits include retaining the bonding characteristics of films and adhesives, while anti-blocking properties can also be achieved in decorative coatings [150,151].

One of the earliest applications of styryl and methacrylate functional macromers was for controlling release in adhesive tapes [54]. The investigators showed a correlation between molecular weight and release characteristics—e.g., in butyl methacrylate, acrylic acid, and macromer terpolymers—with macromers of low molecular weight proving ineffective but macromers with molecular weights ≥2000 providing control. The same general chemistry has been utilized more recently in dirt resistant coatings [93]. Release coatings, including ice-phobic coatings, have been generated from carbinol-terminated siloxane macromers by reaction with isocyanate and epoxy functional prepolymers to form amphiphilic, self-stratified thin films [152]. A reduction of marine biofouling was observed when aminopropyl-terminated macromers were incorporated into isophorone diiosocyanate-derived urethanes, with low molecular weight macromers (1000 Daltons) providing more favorable results than high molecular weight macromers [81]. Methacrylate-terminated siloxane macromers with embedded hydrophilicity have also been used in this application [108]. Ultraphobic coatings—i.e., coatings exhibiting both superhydrophobic and oleophobic behavior—in which combinations of epoxy functional siloxane telechelics and macromers are reacted with linear and/or branched polyethylenimine (PEI), have been reported by Soane and Ready [94,107]. Hydride-terminated macromers have been used to modify vinyl POSS structures in slippage coatings for ice-phobic applications [91]

### 2.5. Coatings

#### 2.5.1. Thin Film Silicone Coatings

The inclusion of silicone macromers into the formal polymeric structures of coatings, as distinguished from their use as additives, is a growing area of technology. These applications tend to be associated with release, lubricity, and mechanical properties related to direct physical interactions with humans. Indeed, the sensory appeal of coatings has always been an important driver of consumer applications in which positive tactile interaction is critical to acceptance [87], such as synthetic leather, textile finishes, and hair care.

**Scheme 9:**
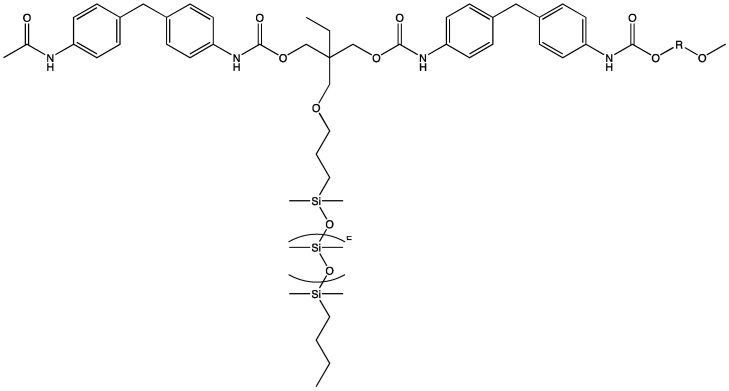
Urethane Polymer with Pendant Siloxane.

**Scheme 10:**
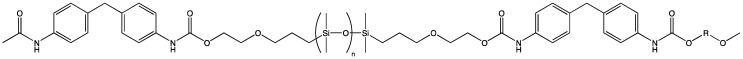
Urethane-Siloxane Soft Segment Block Polymer.

Older approaches to urethane materials mainly use polydisperse telechelic carbinol-terminated siloxane polymers, in which the two identical functionalities on the termini serve to introduce siloxanes into urethanes as soft-blocks. AROP-derived siloxane macromers (oligomeric materials with functionality on one terminus) represent a newer approach in which the functionality on one terminus of the oligomer allows the formation of a brush polymer with siloxane segments as pendant, allowing the mechanical properties of the urethane backbone to be maintained (Scheme 9) [87]. These structures also demonstrate significantly greater wear resistance as well as lower friction and release properties compared to telechelic controls [153], in which siloxane is incorporated into the urethane backbone as shown in Scheme 10. Table 8 compares tribological and contact angle properties of urethane in which siloxane has been introduced as an unreacted fluid, a soft segment, and a pendant, respectively.

In similar chemistry, self-lubricating cannulae for medical applications have been fabricated from thermoplastic urethanes derived from mono dicarbinol macromers [86].

#### 2.5.2. Thick Film Silicone Coatings

**Scheme 11:**
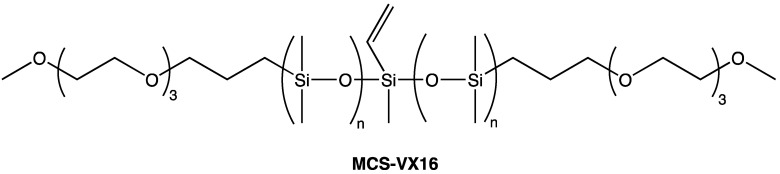
Vinyl functional Siloxane with PEG Endgroups-Reactive in Two-component Pt-Cure Silicone RTVs.

**Scheme 12:**
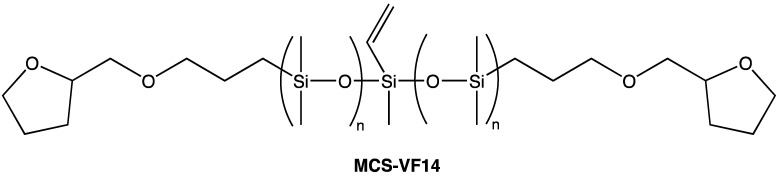
Vinyl functional Siloxane with Tetrahydrofurfuryloxy Endgroups-Reactive in Two-component Pt-Cure Silicone RTVs.

**Figure 7:**
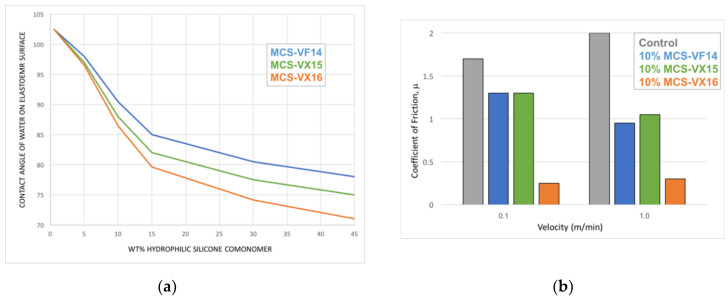
Modification of silicone elastomers with symmetric silicone macromers. (**a**) Effect of comonomer on contact angle and hydrophobicity of silicone elastomer; (**b**) coefficient of friction of silicone elastomers modified with symmetric silicone macromers (Determined on AR-G2 Rheometer: aqueous, 37 °C, normal force 1N, velocity 1.0 rad/s (1.5 mm/s) and 1.0 rad/s (15 mm/s).)

Depending on their formulation, silicone coatings elicit a wide range of tactile responses. Coatings that contain low molecular weight, particularly volatile species are associated with a “silky” feel and exhibit slip. On the other hand, silicone coatings that have been depleted of low molecular weight species are associated with a “tacky” feel. Common to both these experiences is the extreme hydrophobicity of the polymer. When silicone coatings are free of low molecular-weight species, they exhibit high coefficients of friction and, due to their relatively poor mechanical properties, failure by abrasive and adhesive spalling during continuous tactile interaction. Well-defined silicones with a central vinyl functionality and discrete PEG_2_ (MCS-VX15), PEG_3_ (MCS-VX16—Scheme 11), or tetrahydrofurfuryl (MCS-VF14—Scheme 12) pendant end-groups can be used as comonomers in addition-cure, platinum-catalyzed 2-part silicone elastomer formulations in order to introduce hydrophilicity [61]. In such formulations, the surface tribological properties are modified by introducing a hydrodynamic lubricating layer of adsorbed water. The modified silicone elastomers retain optical clarity and mechanical performance characteristic of this class of material with up to 15 wt.% comonomer in the 2-part formulation. Contact angle measurements of deionized water on the silicone elastomer surface showed improved wettability with comonomer content: at ~3 wt.% comonomer, the elastomer surface shifts from hydrophobic (contact angle ~120°C) to hydrophilic (contact angle < 90°C). Coefficient of friction measurements for the modified silicone elastomers demonstrate increased surface lubricity with comonomer loadings (Figure 7) [112].

Separately, in the field of dielectric elastomer actuators, monovinyl-terminated PDMS macromers have been used to selectively adjust the network behavior of silicone films between compliant electrodes [111].

### 2.6. Cosmetics and Hair Care

**Scheme 13:**
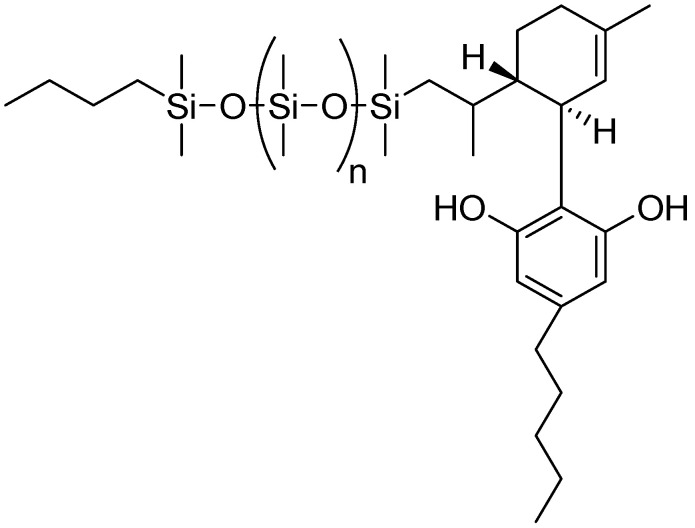
CBD terminated PDMS macromer.

Hair-care formulations, including shampoos and conditioners with the ability to withstand multiple washings, require good film-forming properties with strong adhesion to the hair cuticle, but must simultaneously offer lubricity in order to provide combability. Copolymers of dimethylaminoethylmethacrylate and low molecular weight methacrylate functional silicone macromers (MW 2000) [89,90] have been used directly or in combination with other polymers [154] to provide increased lubricity and combability of hair. Dispersions of macromer-derived terpolymer particles have been reported in hydrocarbon vehicles such as isododecane, yielding film-forming compositions that are useful in eyeliners and mascaras [97]. Hydride-terminated macromers have been reacted with unsaturated terpenes and cannabidiol (CBD) (Scheme 13 to form emollient compounds with solubility in the siloxane vehicles preferred for skin care [155,156].

### 2.7. Magneto-Rheological Fluids

**Figure 8:**
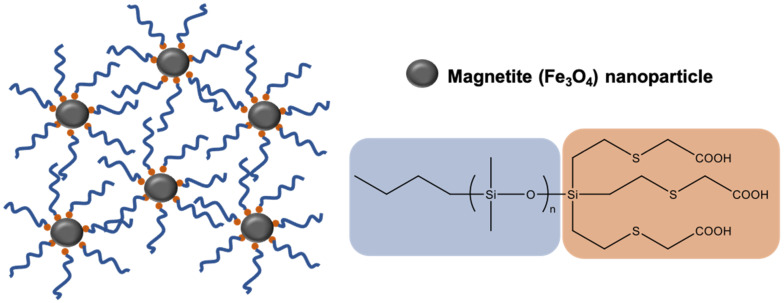
PDMS macromer-modified SPIONs (Author’s work).

Well-defined biocompatible magnetic nanoparticles are of interest as materials for biomedical applications including magnetic field-directed drug delivery, biomolecule separations, and assay devices. Superparamagnetic iron oxide (Fe_3_O_4_) nanoparticles (SPION) sterically stabilized with PDMS macromers synthesized by Living AROP produced homogeneous hydrophobic ferrofluids that are stable against precipitation [157,158]. PDMS macromers with a tricarboxylate endgroup capable of binding to the surface of magnetite nanoparticles were synthesized by first making a trivinyl-terminated PDMS via Living AROP, followed by a thiol-ene reaction between the vinyl silane groups and mercaptoacetic acid, as depicted in Figure 8 [159,160]. Molecular effects of the PDMS tail on the stability of the PDMS-magnetite complexes were studied. Magnetic separation methods were developed to narrow the particle size distribution of the magnetite nanoparticles using tricarboxylate PDMS stabilizer while controlling the PDMS surface concentration [161]. In other studies, a monocarboxydecyl-terminated PDMS macromer (MCR-B12) was used to stabilize magnetite nanoparticles; however, the resulting ferrofluids had issues with stability and sedimentation, likely due to the lower number of carboxylate binding groups per macromer chain [88]. The magnetophoretic mobility of the magnetite-PDMS fluids was then studied in different magnetic field conditions (magnetic fields and field gradients), with the results demonstrating that the shape and speed of these droplets in viscous media can be independently manipulated [160,162].

**Figure 9:**
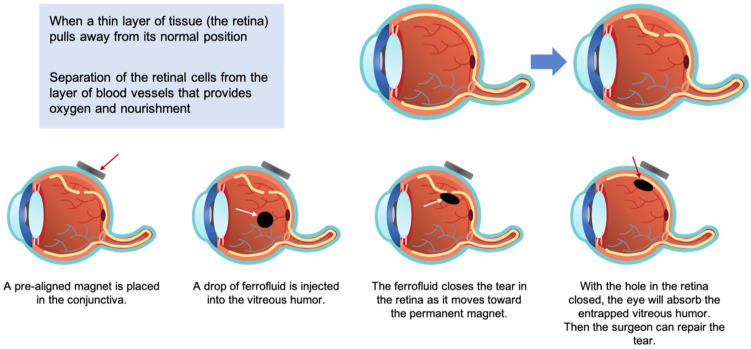
Retinal repair utilizing macromer-enabled magnetorheological fluids.

These magnetite-polydimethylsiloxane ferrofluids were proposed by Wilson as a material that could aid in the treatment of retinal detachment disorder [159]. The proposed treatment entailed inserting a pre-aligned magnet into the conjunctiva and then injecting the PDMS-based ferrofluid into the vitreous humor. As shown below in Figure 9, the ferrofluid would close the tear as it moved toward the permanent magnetite, allowing the surgeon to repair the tear.

### 2.8. Bulk Macroscale Materials

Ultra-High Elongation Elastomer

**Scheme 14:**
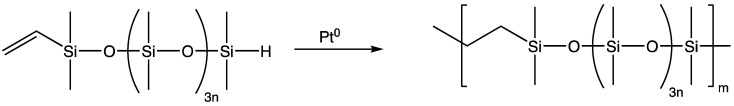
Step-Growth Polymerization of Heterobifunctional α-Vinyl-ω-Hydride-terminated Silicone Macromer.

An ultra-high elongation silicone elastomer has been prepared from a heterobifunctional silicone macromer compounded with reinforcing agent, achieving elongations nearing 5000%, nearly four times greater than conventional silicone elastomers. The cure mechanism of this elastomer is a step-growth polymerization of an α-vinyl-ω-hydride-terminated silicone macromer [62,113,114] via intermolecular hydrosilylation reaction, which yields a linear polymer of exceptionally high molecular weight with no apparent covalent crosslinking (Scheme 14) [73,113].

**Figure 10:**
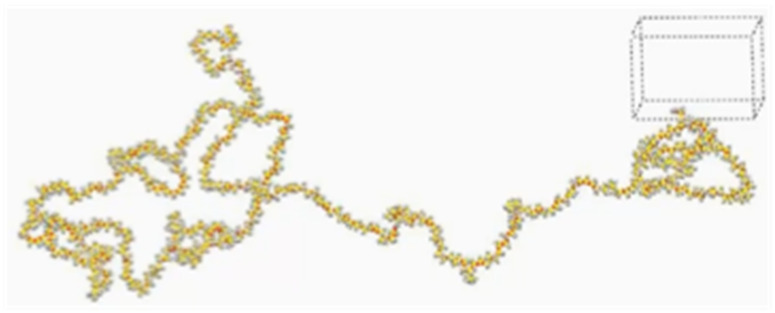
Atomistic model of a 50,000 Da segment of ultra-high elongation elastomer suggesting 1–2 knots. (Author’s work).

**Figure 11:**
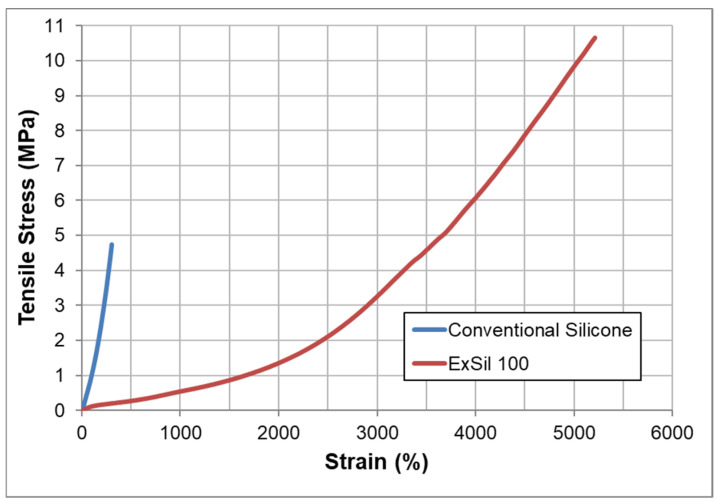
Mechanical properties of ultra-high elongation elastomer (ExSil 100) compared to conventional silicone elastomer.

Atomistic modeling (Figure 10) of the cured silicone macromer shows the probability of knotting within a 50,000 Da segment [115], which correlates to an experimental value of critical molecular weight (M_c_) for entanglement of ~42,000 Da [163]. The stress-strain curve is remarkably different from those of crosslinked silicone elastomers, as shown in Figure 11.

**Figure 12:**
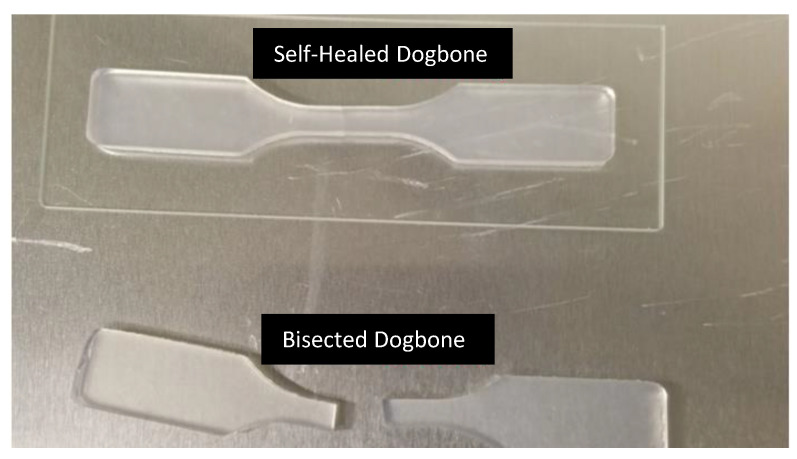
Pseudo-self-healing demonstration of bisected specimen of ExSil^®^ (Author’s work).

Ultra-high elongation elastomers exhibit other behaviors not shared by conventional silicone elastomers [115]: e.g., they undergo extreme multi-axial distortions and return close to their original shape, and display remarkable tear propagation mechanisms, meaning that tear failure occurs at drastically greater elongations; recovery from minor penetration is improved below the failure limits and, finally, pseudo-self-healing behavior is also displayed, as shown in Figure 12 [116].

### 2.9. Nanoscale Morphology

#### 2.9.1. Photoresist and Contact Printing

**Figure 13:**
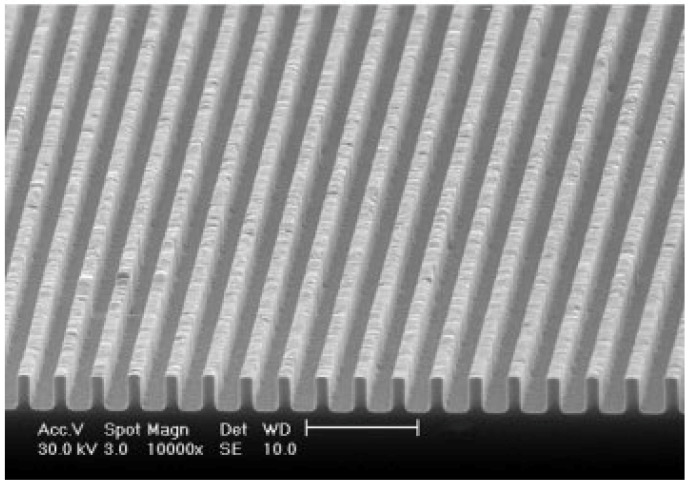
PS-b-PDMS after a single imprint cycle and annealing provides 60 nm features (Reprinted with permission from Ref. [173] Copyright 2007 Wiley).

**Scheme 15:**
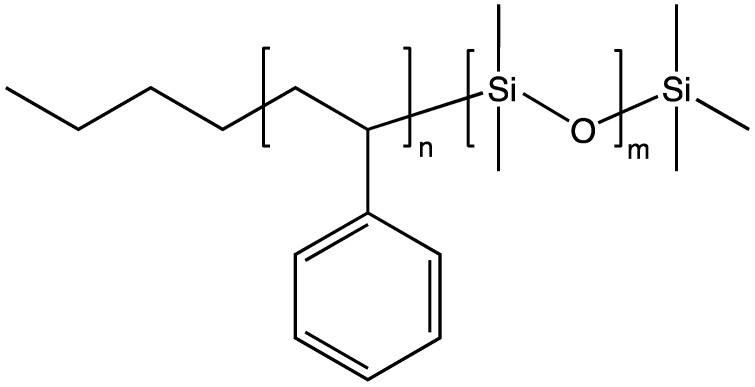
Polystyrene-Polydimethylsiloxane Block Copolymer.

Templated self-assembly of a cylinder-forming poly(styrene-b-dimethylsiloxane) (PS−PDMS) diblock copolymer was first described by Saam and Fearon [51]—followed by others [164,165,166,167]—and has been investigated for nanolithography applications [44,145,168,169]. The general structure is depicted in Scheme 15. The general structure (PS−PDMS) diblock copolymer is depicted in structure 11. These copolymers undergo microphase segregation above their *T_g_*, and the large X-polymer-solvent interaction parameter of the blocks is advantageous for achieving long-range ordering as well as for minimizing defect densities. Furthermore, the high Si content in PDMS leads to a robust oxide etch mask after two-step reactive ion etching (RIE) [170], as exemplified in Figure 13.

To address the critical needs of nano-dimensional photoresists, materials that possess dual surface properties are required. When cast or hot-pressed on a high-surface-energy substrate such as silicon, glass, or aluminum, the copolymer film forms both a lower-surface-energy component (PDMS)-enriched air/polymer interface and a higher-surface-energy component (organic block)-dominated polymer/substrate interface [171,172,173].

Interface effects complicate the use of siloxane A-B block copolymers due to the low surface energy and wetting characteristics of the siloxane block.

Silylated styrene block polymers that employ conventional radical polymerization rather than AROP have been proposed [174,175], and azidopropyl functional silicone macromers have been separately “clicked” with alkynyl-terminated ATRP-generated macromers to form PDMS-b-PMMA, producing sub-10 nm structures [174,176]. A second potential solution is star block polymers generated by AROP, which provide a mechanism through which the non-siloxane block can dominate interfacial behavior [52]. The reviewers note that similar morphologies have also been reported for polybutadiene-polydiethylsiloxane block copolymers (PBD-b-PDES) [19]; these copolymers possess less differentiation in surface energy, potentially mitigating issues with the styrenic and methacrylate systems, but have not been evaluated in lithographic systems. Similarly noteworthy in this context, while the synthesis of diblock polymers typically starts with an organic macroanion and then proceeds to a siloxane polymerization, the potential of the reverse process, in which a siloxane macromer starts and proceeds to an acrylate polymerization, was demonstrated using ATRP [177]. Other organic block copolymer polymerization examples initiated by carbinol-terminated macromers include caprolactone and trimethylene carbonate blocks [83,178]. This approach clearly expands the options for generating polymers with varying self-assembly structures.

**Figure 14:**
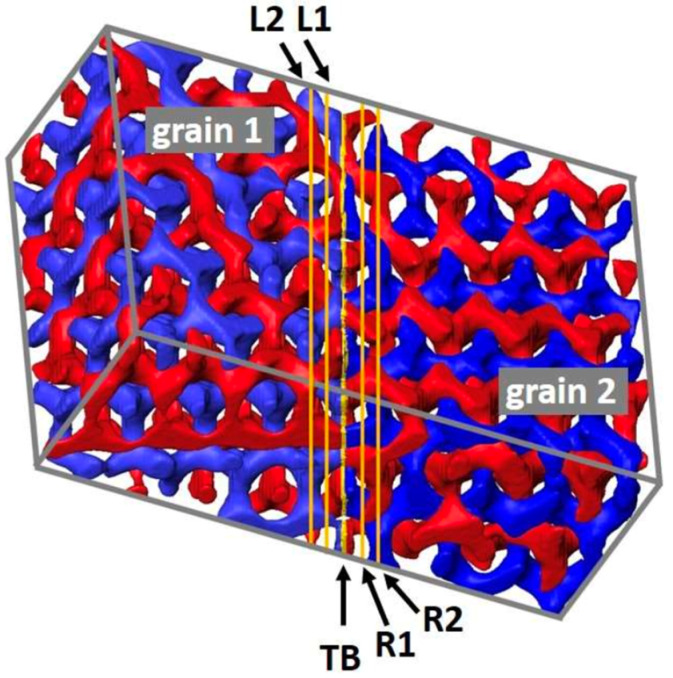
Reconstruction of a volume containing a Double Gyroid of PS-b-PDMS (Reprinted with permission from Ref. [183] Copyright 2021 National Academy of Sciences).

The use of polystyrene macroanions was further elaborated by Bellas to form triblock and microarm polymers [44], and by Shefelbine to form continuous core–shell gyroid morphologies [179]. This work led to the observation of periodic double gyroid (DG) behavior, as well as a series of publications displaying an appreciation of the potential for extended novel 3D structures (Figure 14) [180,181,182]. As suggested by the DG topological visualizations of Thomas [183], potential structural, dielectric, charge transport, and mass transport material behavior can be controlled by the direct use of DG block polymers structures or by the removal, conversion, and/or infiltration of a DG microphase.

#### 2.9.2. Biomimetic Polymers and Bottle-Brush (BB) Architecture

Recently, grafting-through polymerization and surface-initiated polymerization have led to bottle-brush polymers and particle-brush materials that have shown potential in the fabrication of biomimetic materials. These materials can be broadly considered filamentous structures that exhibit non-linear behavior under deformation, greater relaxation times, and the potential for complex non-covalent interactions leading to the formation of super-molecular structures. Siloxane macromers appear to be of particular interest in creating these structures due to both the palette of functionality and the intrinsic flexibility of the siloxane structures, which allows the length-scale of the filaments to extend beyond the primary polymer backbone or nano-feature without imposing a “hard” structural domain. Siloxane macromers allow entry to classes of materials that possess an unusually low modulus while maintaining mechanical failure properties consistent with the main polymer backbone for both methacrylate [96] and norbornene [84] functional siloxane macromers.

**Figure 15:**
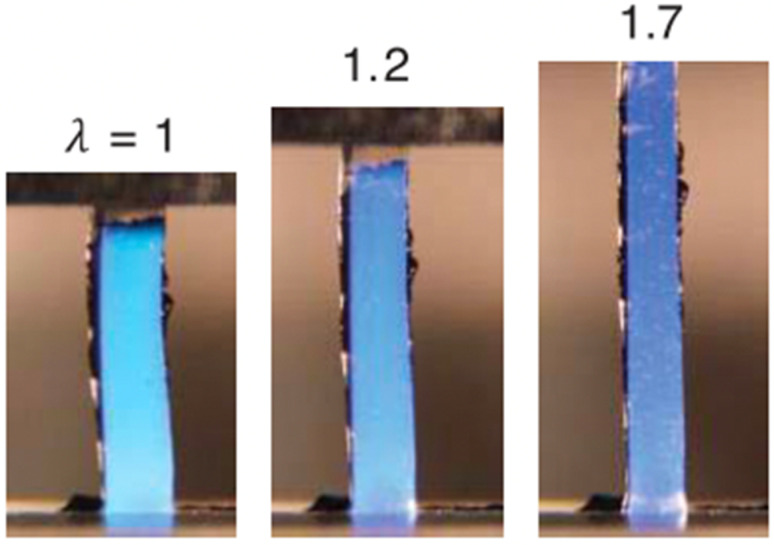
Color alteration from turquoise to dark blue during uniaxial stretching. (Reprinted with permission from Ref. [106] Copyright 2018 AAAS).

Separately, this recognition revealed such macromers’ potential for generating biomimetic gel structures [95]. The complexity of the potential range and behavioral characteristics associated with bottle-brush polymers in terms of macromer molecular weight, graft density, and final molecular weight is readily apparent. Their tunable physical properties in both crosslinked and uncrosslinked states—based on the Dp of the main chain and grafting density of methacryloxypropyl-terminated polydimethysiloxane (Dp = 10) prepared by Grafting Through Atom Transfer Radical Polymerization—have been investigated in this context [103]. The dynamics of deformation have also been modeled [104,109,110]. A striking extension of this approach can be found in the “chameleon-like” color changes demonstrated in these systems—in which vibrant color, extreme softness, and intense strain stiffening on par with that of skin tissue have been observed as a consequence of placing a heterogeneous polymeric system under varying degrees of strain (Figure 15) [106].

#### 2.9.3. Liquid Crystal Siloxanes

**Figure 16:**
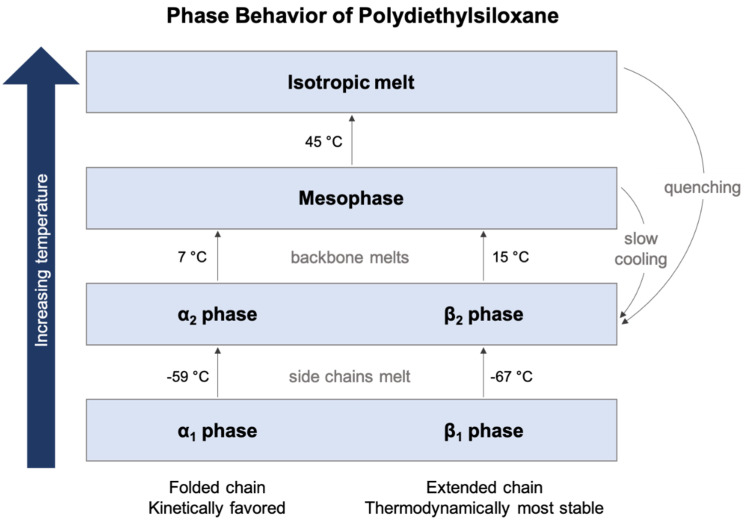
Liquid Crystal-Phase Behavior of Polydiethylsiloxane.

Most polymers that exhibit liquid crystalline behavior have anisotropic side groups. Polydiethylsiloxane, which is distinct from both lost LCPs and its simpler homolog, polydimethylsiloxane, demonstrates mesophasic liquid crystalline behavior at ambient temperatures, as first reported by Beatty [184]. This behavior extends to poly(di-n-alkylsiloxane)s with side chains no longer than seven carbons that are also able to form a columnar mesophase. Such polymers are positionally and orientationally ordered in a two-dimensional hexagonal lattice, but without positional order along the chain. While early work was conducted with polymers of high polydispersity (>2.0), the desire to optimize liquid crystal behavior by controlling the PDI has led to the use of living AROP conditions for the polymerization of polydiethylsiloxane (PDES)—as first reported by Molenberg, who utilized lithium sec-butyldiethylsilanolate as the initiator [41,43]. The phase behavior of diethylsiloxane is shown in Figure 16.

There are few reports of living AROP with higher dialkylsiloxanes, presumably because of either ineffective termination or these systems’ sluggish kinetics.

**Figure 17:**
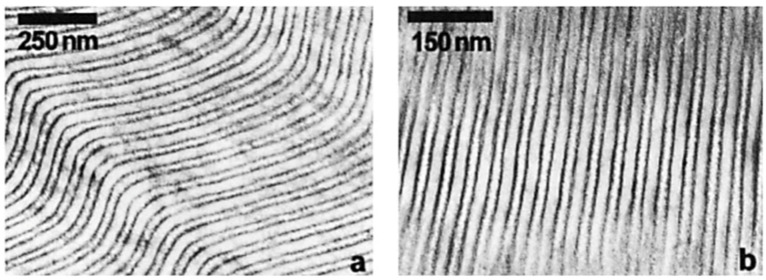
Bulk structures of PolyStyrene-Polydiethylsiloxane block copolymers. Bulk structures of (**a**) PS-*b*-PDES containing 37 wt% PS and (**b**) PS-*b*-PDES containing 43 wt% PS (Reprinted with permission from Ref. [19] Copyright 1998 Wiley).

Molenberg also reported on elastomeric block polymers of butadiene and diethylsiloxane that exhibited mesophase formation under tensile stress [19]. Later work with styrene-diethylsiloxane diblock polymers showed periodic nanoscale lamellar structures with compositions possessing greater than 20% styrene content, as shown in Figure 17 [42].

**Scheme 16:**
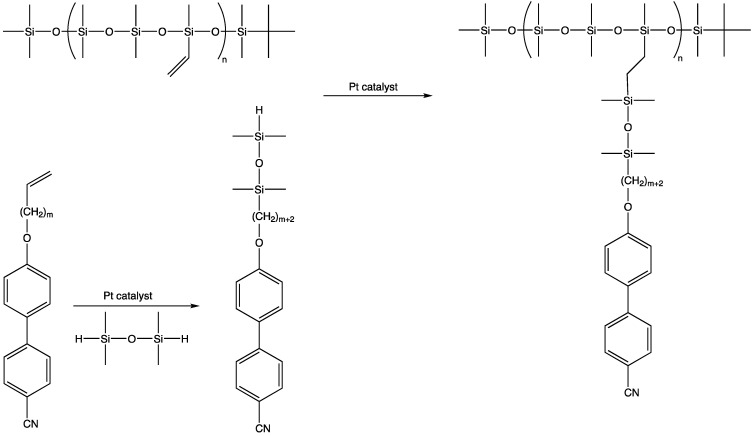
Derivatization of AROP Siloxanes with mesogenic pendant groups.

The vast majority of liquid crystal polysiloxanes based on side chain substitution that have recently been reviewed are polydisperse in nature [185]. Hepenius, however, utilized AROP to form vinylmethylsiloxane copolymers from vinylpentamethylcyclotrisiloxane and then functionalized the positions with mesogenic groups: for example, by reacting 4-cyano-4′-(ω-alkenoxy)biphenyl with an excess of tetramethyldisiloxane and then reacting with the remaining Si-H group (Scheme 16) [46].

## 3. Conclusions

The accelerating interest in living AROP-derived siloxanes has been spurred by the increasing drive to provide soft matter structures associated with tactile interaction and biocompatibility, particularly in the areas of contact lenses and biomimetic structures, and by these materials’ ability enable control of nano-dimensional physical properties associated with “smart” particles and morphologies associated with self-assembly. Silicone macromers have had great impact in practical applications, primarily due to their ability to cross-over from the paradigm of inorganic siloxane chemistry to the greater paradigm of organic polymerization. Further, the elaboration of simple macromers into heterobifunctional monomers has created opportunities for polymers with new bulk phase properties, including ultra-high elongation and self-healing materials.

## Figures and Tables

**Table 1:** Cyclic Siloxane Macromer “Building Blocks”.

Cyclic Siloxane Macromer Monomers					
Class	Structure	MW	B.P.	CAS#	References
**Primary**					
Hexamethylcyclotrisiloxane	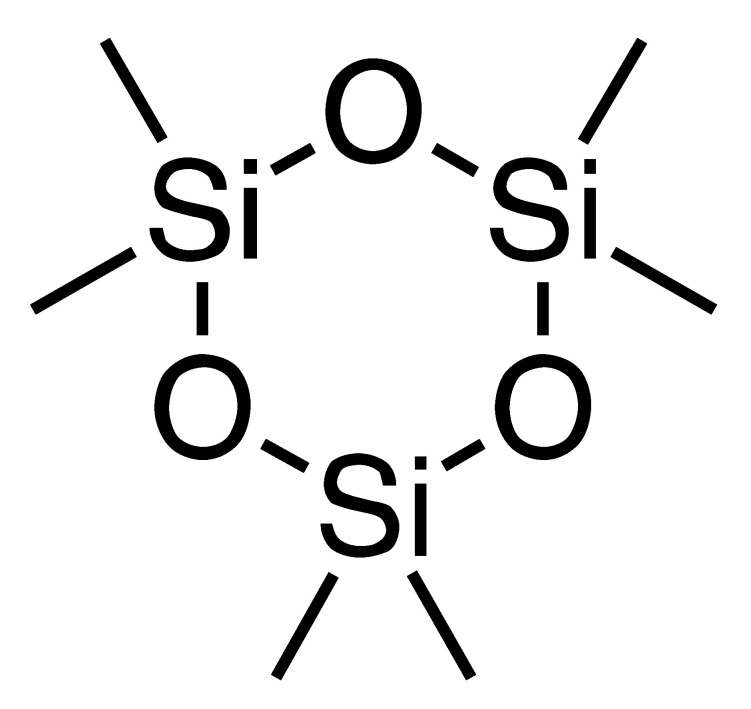	222.46	134°	541-05-9	Hyde [16]
Hexaphenylcyclotrisiloxane	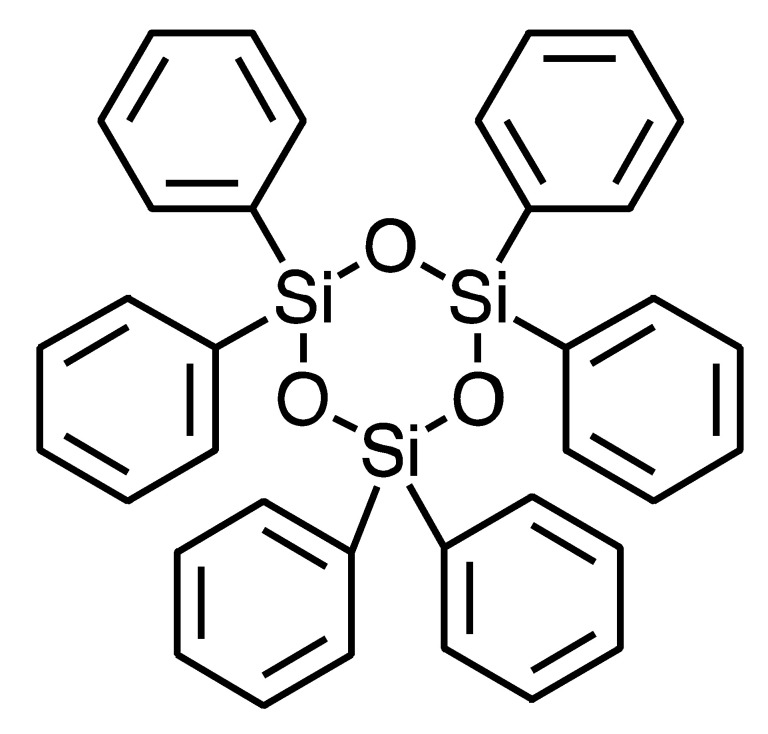	594.89	300°/1	512-63-0	Bostick [32]
Trivinyltrimethylcyclotrisiloxane	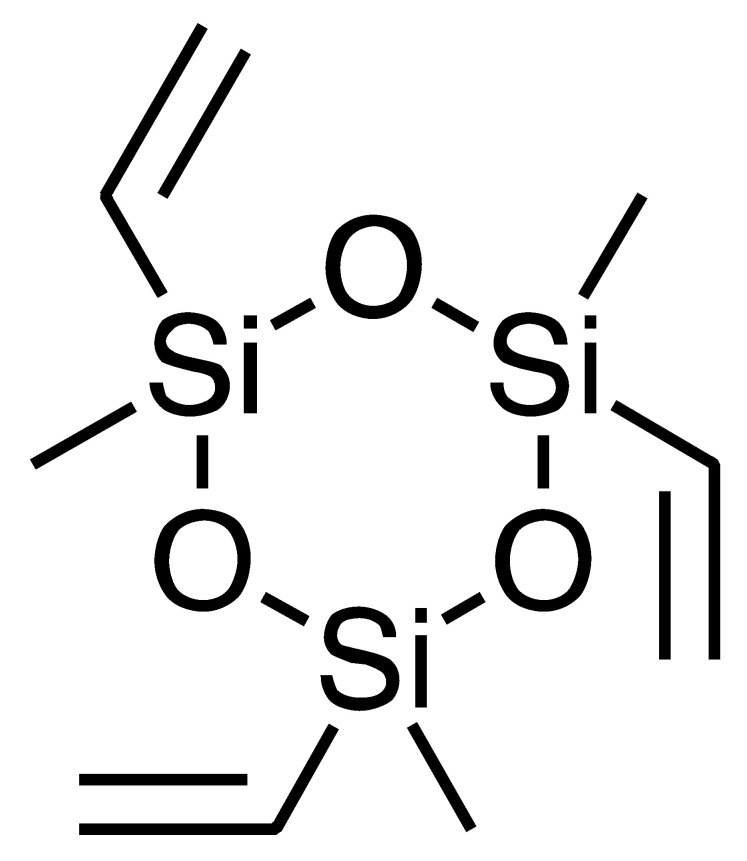	258.50	80°/20	3901-77-7	Pike [33]
Tris(3,3,3-trifluoropropyl)trimethylcyclotrisiloxane	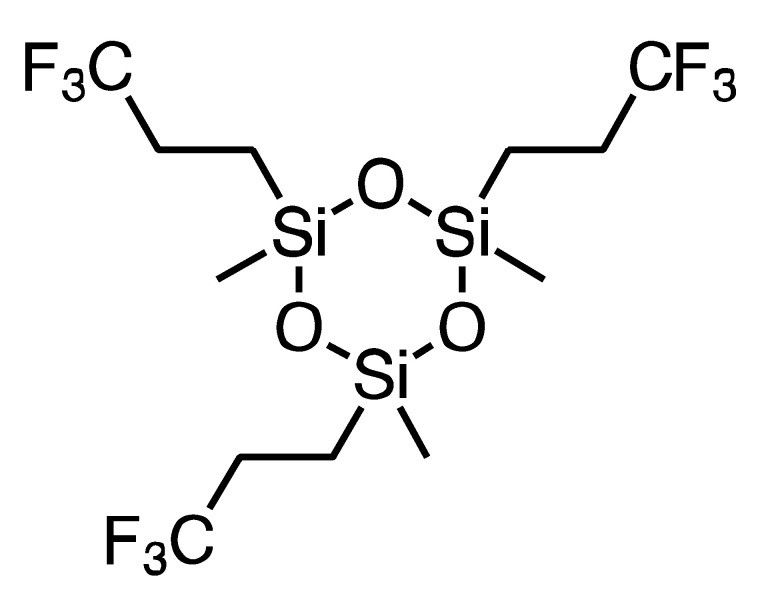	468.55	95°/3	2374-14-3	Pierce [18]
2,2,5,5-tetramethyl-2,5-disila-1-oxacyclopentane	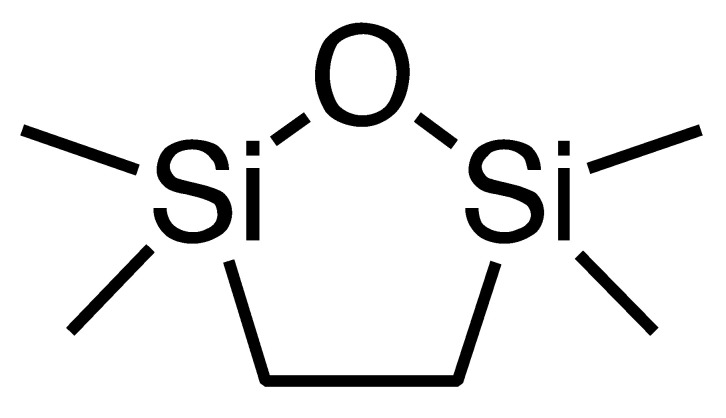	160.36	124°	7418-20-4	Piccolli [17]
**Minor**					
Hexaethylcyclotrisiloxane	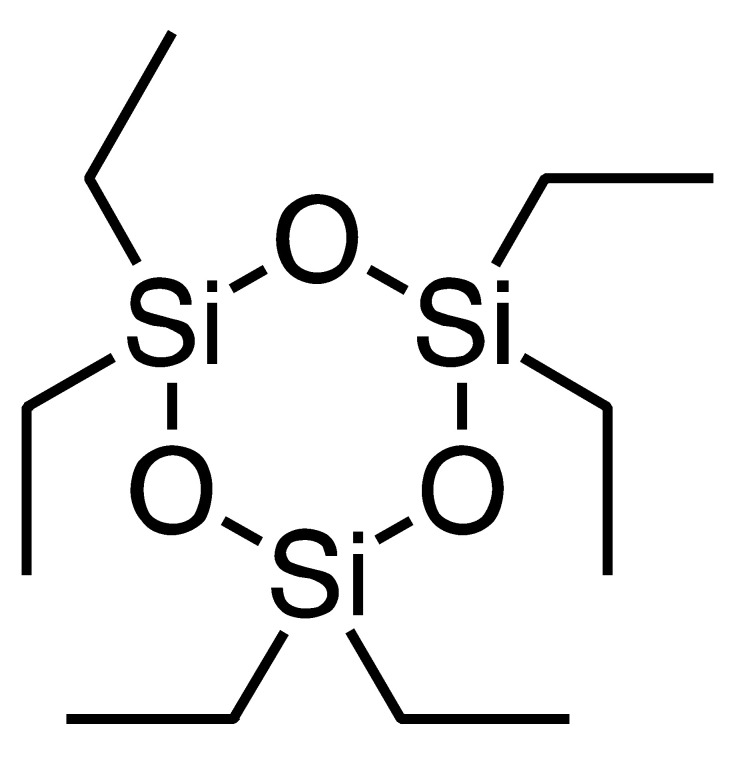	306.82	117°/10	2031-79-0	Dobay [34]
Vinylpentamethylcyclotrisiloxane	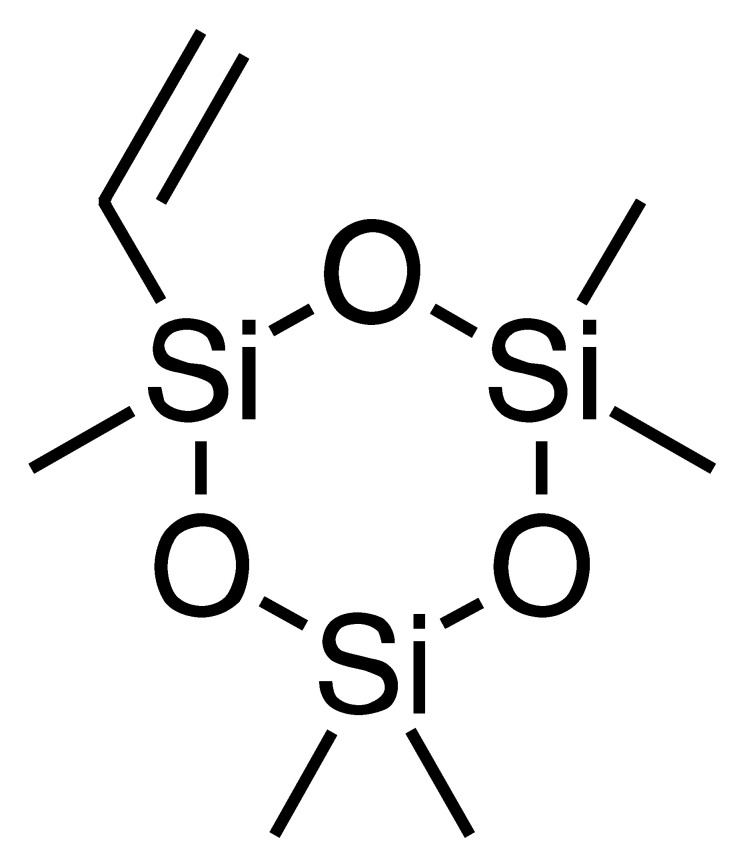	234.47	56°/23	18395-32-9	Rózga-Wijas [23]
Tris(tetramethylsiloxanylethyl)trimethylcyclotrisiloxane	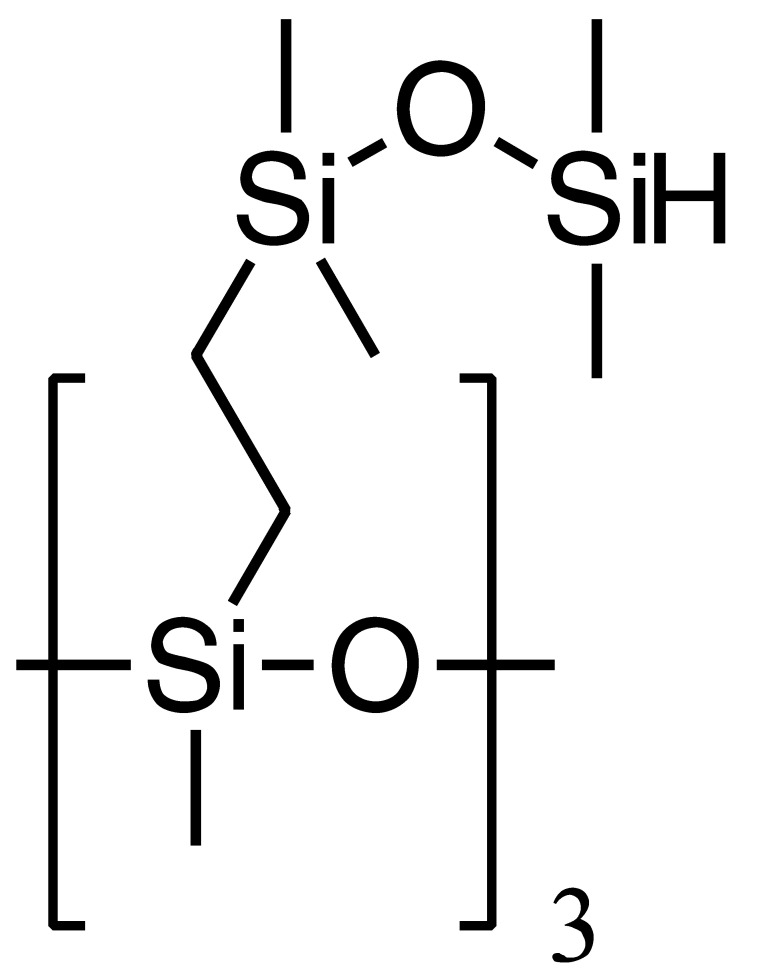	661.47	175°–185°/2	2378614-30-1	Goff [22]
1,3,5-[Tris(methoxyethoxyethoxypropyltetramethyldisiloxanyl)ethyl]-1,3,5-trimethylcyclotrisiloxane	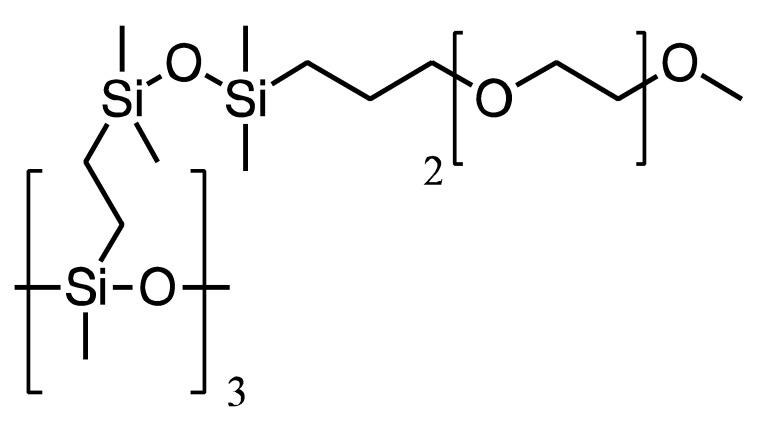	1142.10		2378623-20-0	Goff [35]

**Table 2:** Initiators.

Non-Functional Initiators			
Class	Structure	Comments	References
**Organolithium**			
n-Butyl lithium	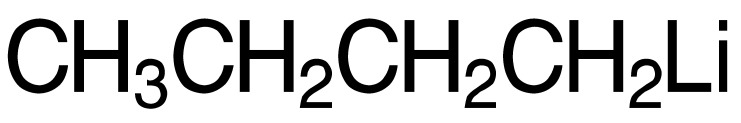	Most common initiator; stable and economic	Chojnowski [36] Cypryk [37] Maschke [38] Nakano [11] Razzano [39]
s-Butyl lithium	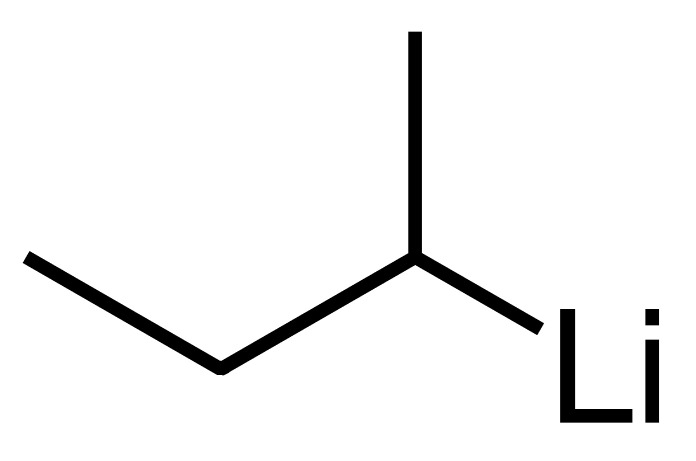	Less initial back-biting than n-butyl but less stable; used for substituted aryl-cyclic trisiloxane; used for higher alkylcyclo-trisiloxanes	Hawkridge [40] Bellas [41] Ninago [42] Lee [26] Molenberg [43,44,45]
t-Butyl lithium	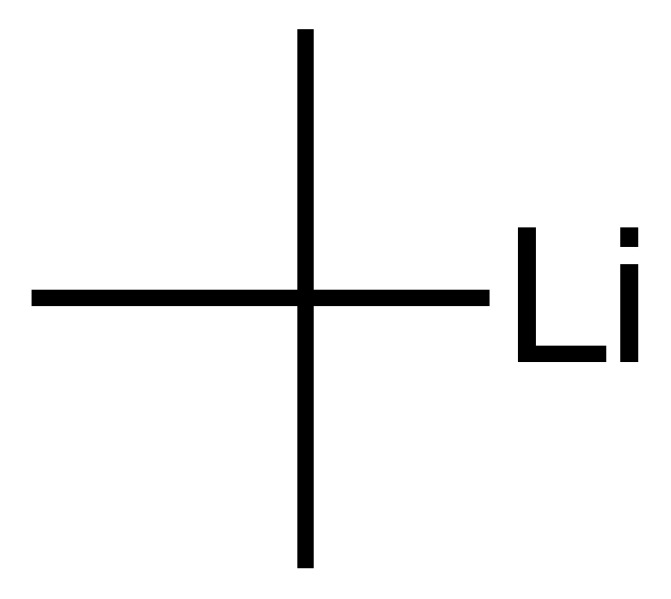		Hempenius [46] Sieburth [47]
Phenyl lithium	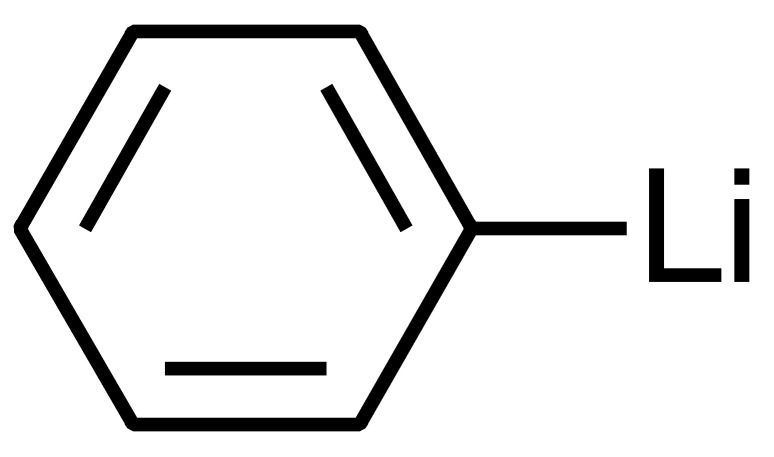	Initiator for PDMS MW standard	Arkles [48]
Trimethylsilylmethyl lithium	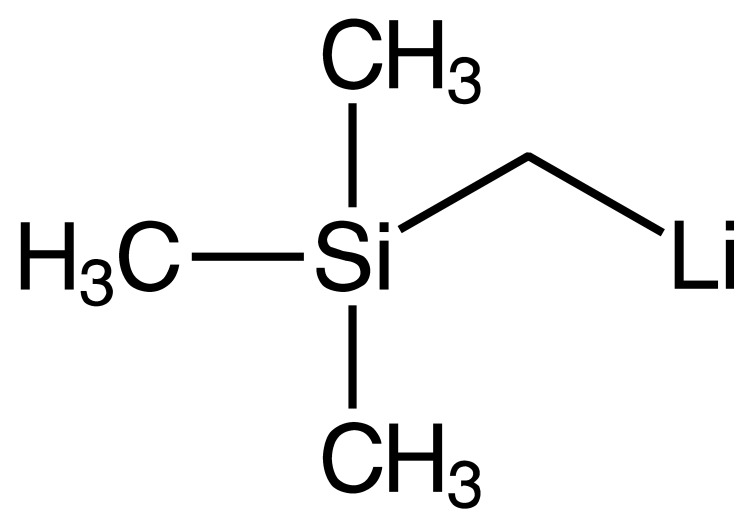	For diMeSiO-ViMeSiO block polymers	Boileau [49] Bauer [50]
**Macroanions**			
Polybutadienyl lithium	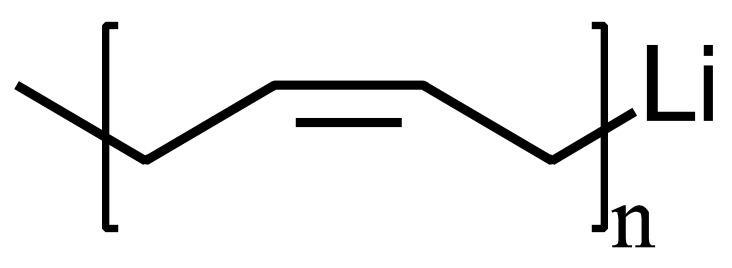		Molenberg [19]
Polystyryl lithium	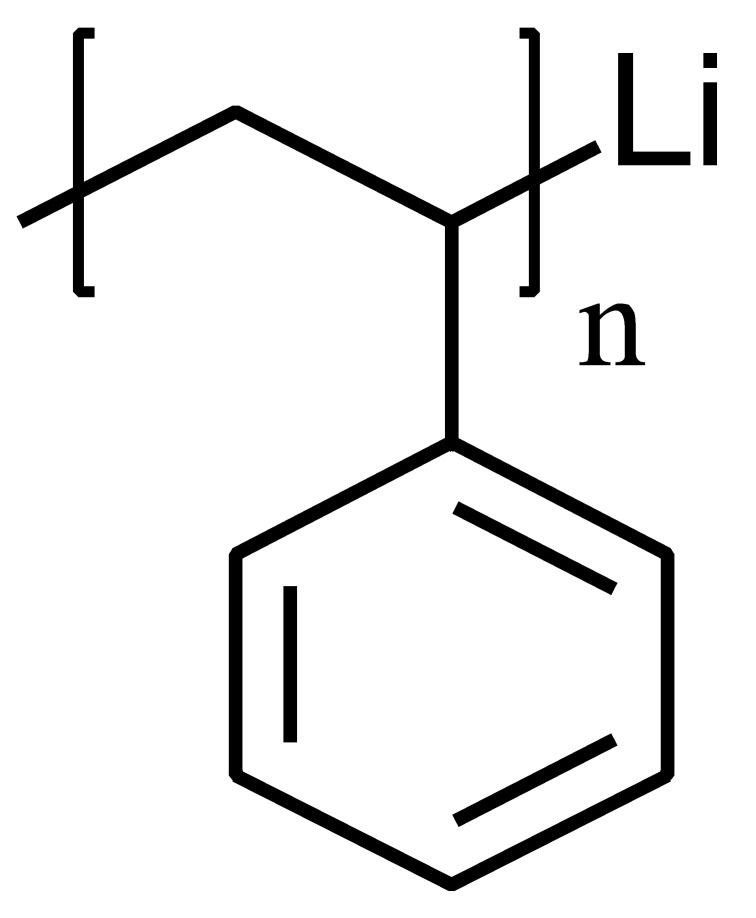	Formation of block and star polymers—varied block size for nanopatterning	Saam [7,51] Bellas [44] Georgopanos [52]
Poly a-methylstyryl lithium	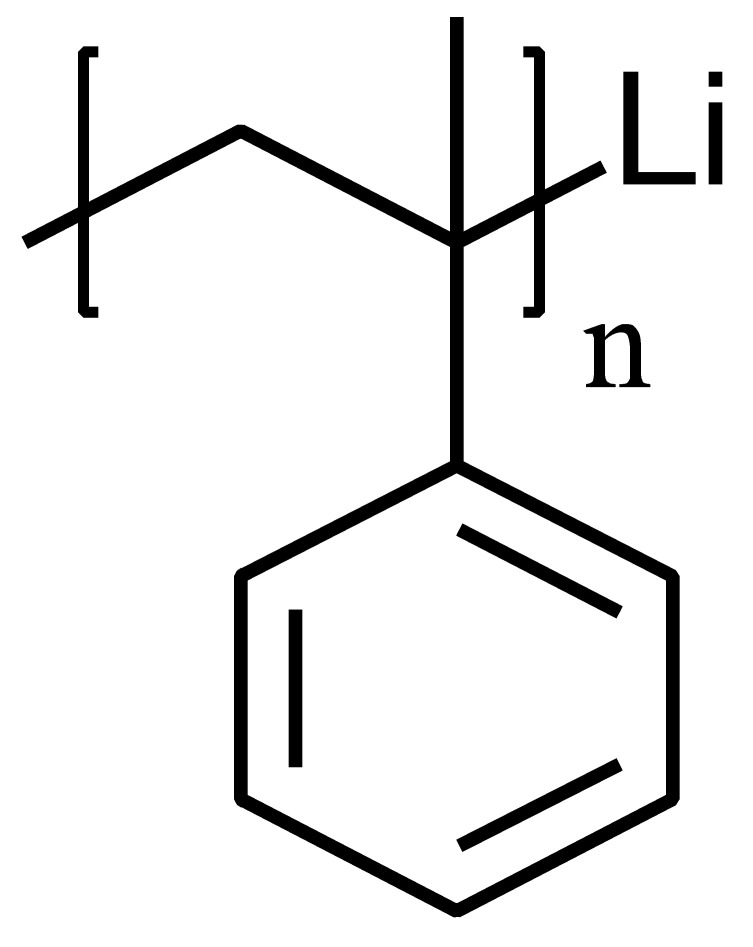		Saam [8] Noshay [53]
**Lithium Silanolate—Monoanion**			
Trimethylsilanol lithium salt	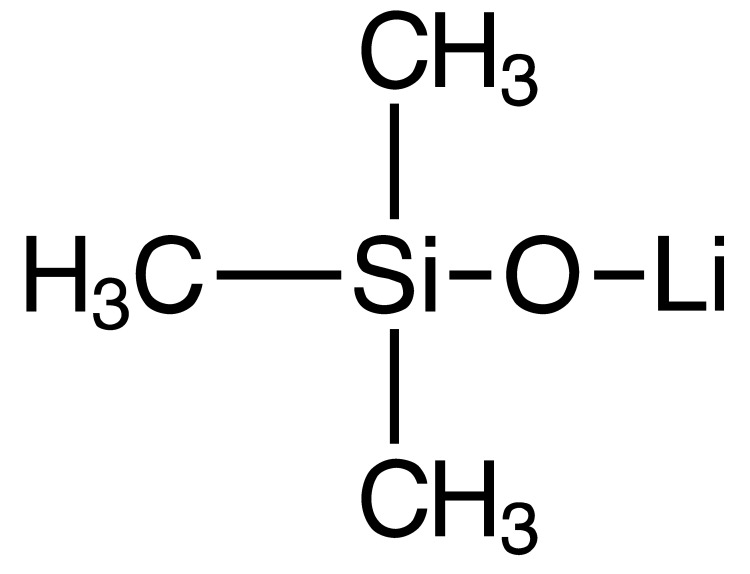	Formed from D_4_ + Methyllithium; initiates tetramethyldisila-oxacyclopentane polymerization	Clemens [54] Kawakami [9] Awasthi [55]
*n*-Butyldimethylsilanol lithium salt	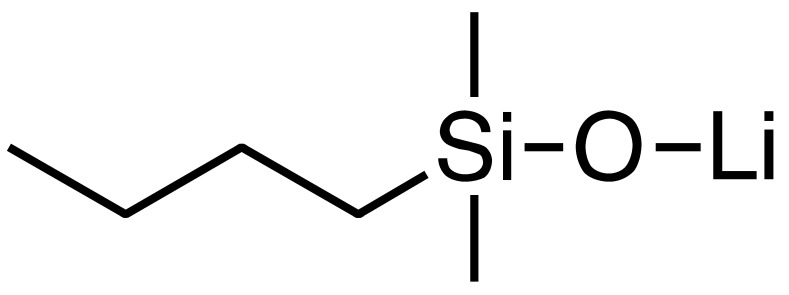	Formed from D_4_ + n-Butyllithium	Fessler [6] Bostick [32] Cypryk [56] Leir [57]
Fluoroalkyldimethylsilanol lithium salt	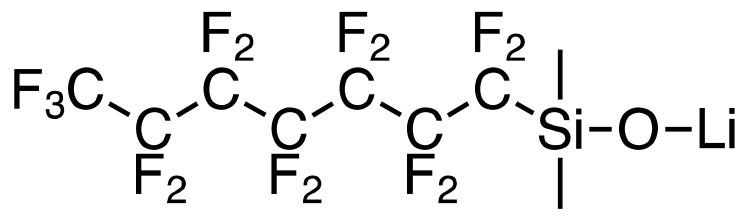	Formed in situ from silanol and n-Butyllithium	Saho [58]
Methoxypropyldimethylsilanol lithium salt	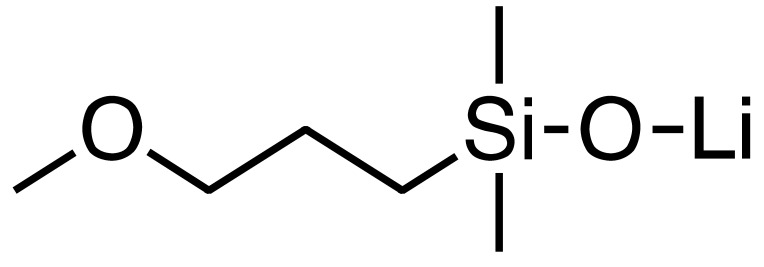		Arkles [59] Goff [60]
Methoxyethoxyethoxypropyldimethylsilanol lithium salt	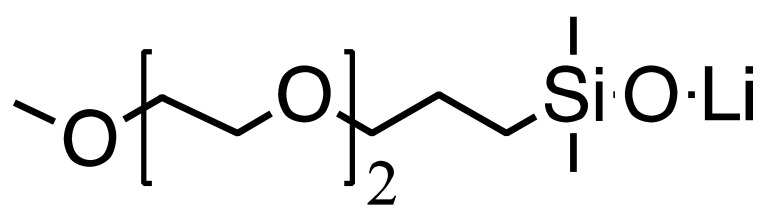	Hydrophilic silicone macromers for contact lenses	Goff [61] Arkles [62]
Methoxyethoxyethoxyethoxypropyldimethylsilanol lithium salt	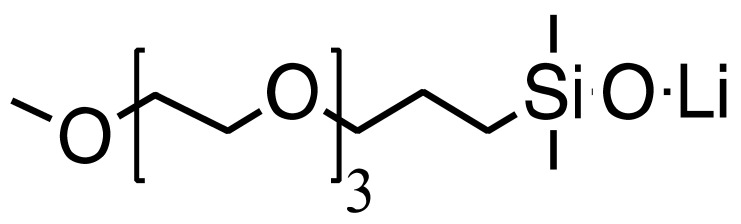	Hydrophilic additive for silicone elastomers	Goff [61] Arkles [62]
Tetrahydrofurfuryloxpropyldimethylsilanol lithium salt	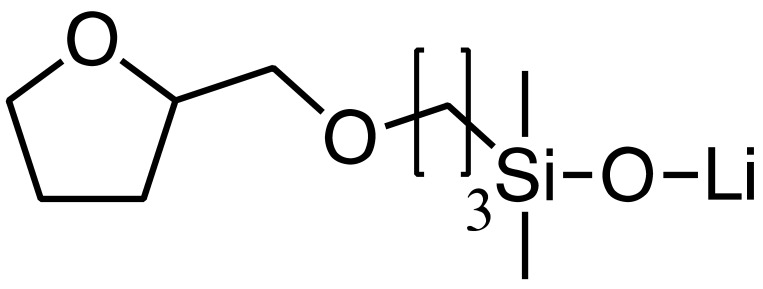	Hydrophilic additive for implantable silicones	Goff [61] Arkles [62]
**Lithium Silanolate—Multiple Anions**			
Diphenylsilanediol, dilithium salt	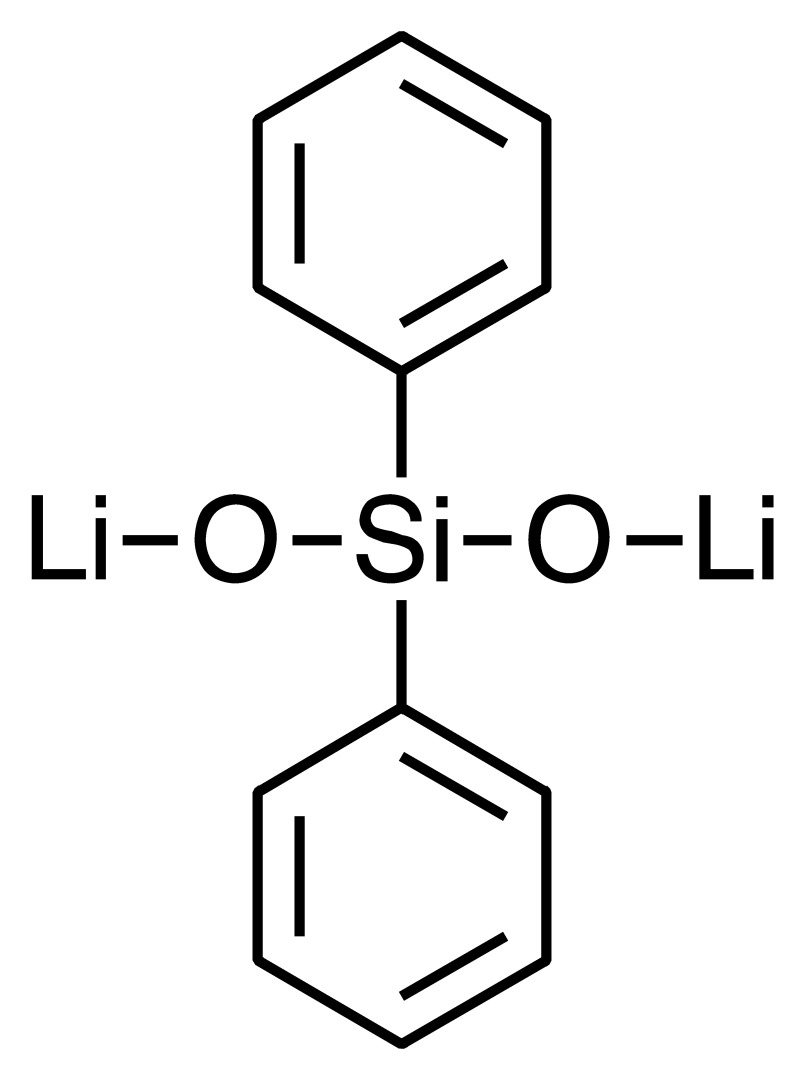	Initiator for pentamethylcyclo trisiloxane; initiator for trifluoropropyltrimethylcyclotrisiloxane	Paulasaari [21] Cypryk [37]
Tetramethyldisiloxane-1,3 diol dilithium salt	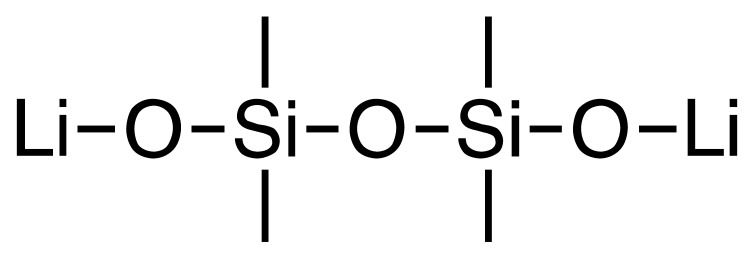		Gadda [63]
1,3-diphenyl-1,3-dimethylsiloxanediol, dilithium salt	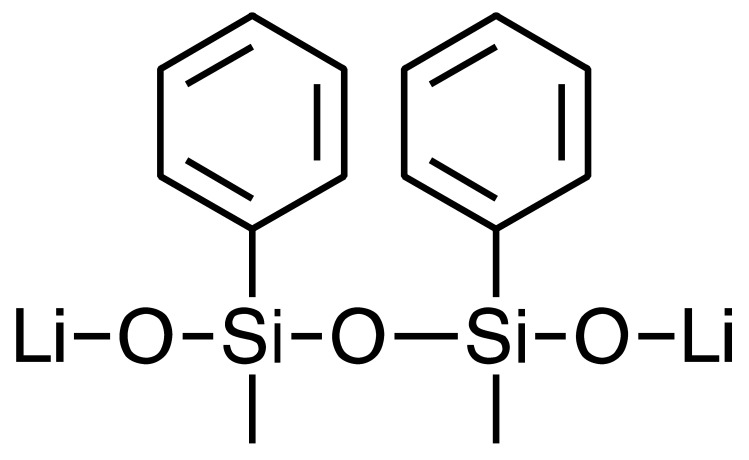		Bostick [32]
Bis(p-hydroxydimethylsilyl)phenyl ether, dilithium salt	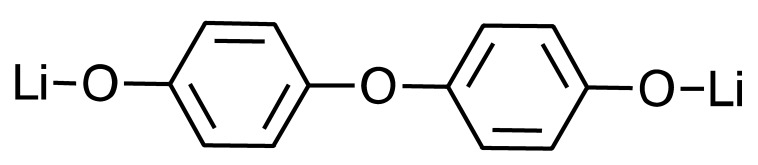	Used to form telechelic vinyl-terminated polymers	Kazama [64]
Branched siloxanes, multiple lithium salts		Branched vinyl-terminated siloxanes	Ogawa [65]
Branched carbosilanes, multiple lithium salts		Used to form multi-arm and dendrimer-like structures	Novozhilov [66] Vasilenko [67]
**Other**			
Dilithiobenzophenone	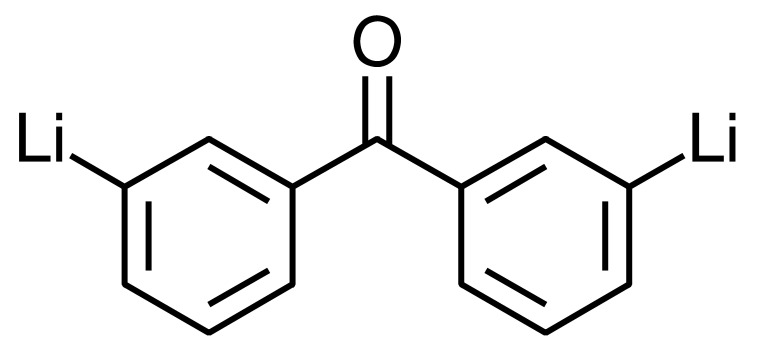	Siloxane methacrylate copolymers	Juliano [68]
Dilithiofluorenone	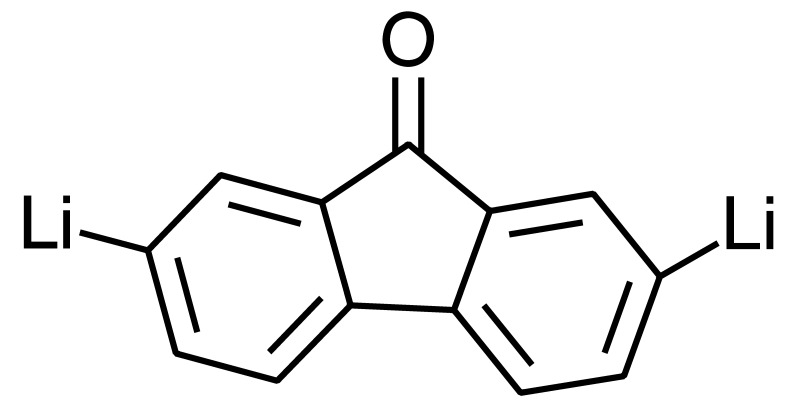	Color change when D_3_ polymerization initiated	Juliano [68]
**Functional Initiators**			
**Class**	**Structure**	**Comments**	**References**
**Organo Lithium**			
Allyl lithium	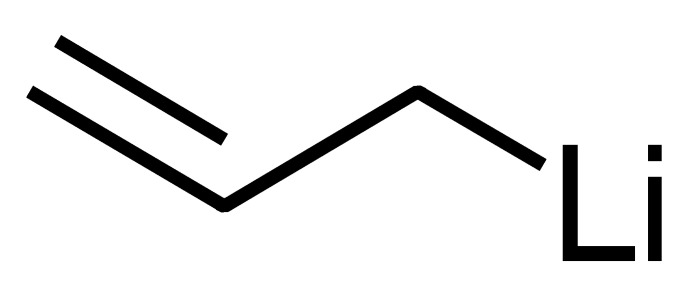	Functionalized polydiethylsiloxane	Out [69]
t-Butyldimethylsiloxypropyl lithium	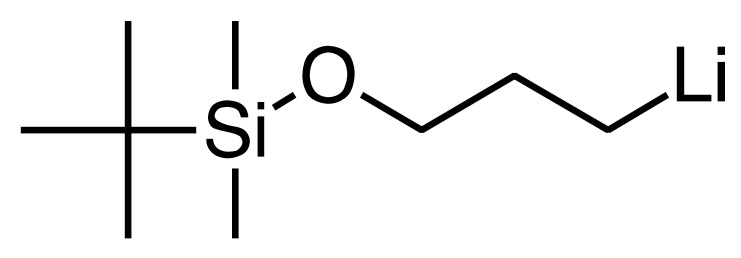	Deprotected to form hydroxyl termination	Arkles [70] Arkles [59]
N-Benzyl-N-methylaminopropyl lithium	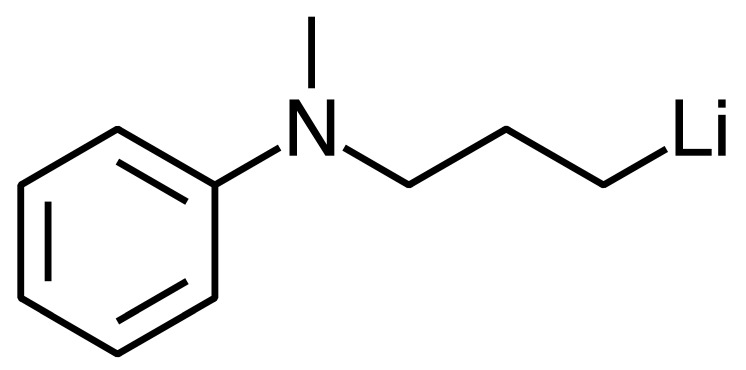	Can be deprotected to provide N-methylaminopropyl termination	Elkins [71]
Bis(trimethylsilyl)aminophenyl lithium	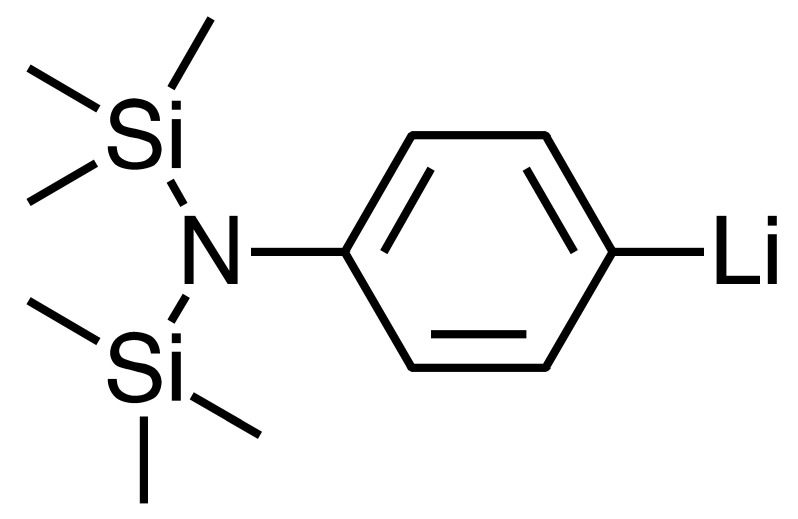	Deprotected to form aminophenyl termination	Babu [72]
**Lithium Silanolate**			
Vinyldimethylsilanol lithium salt	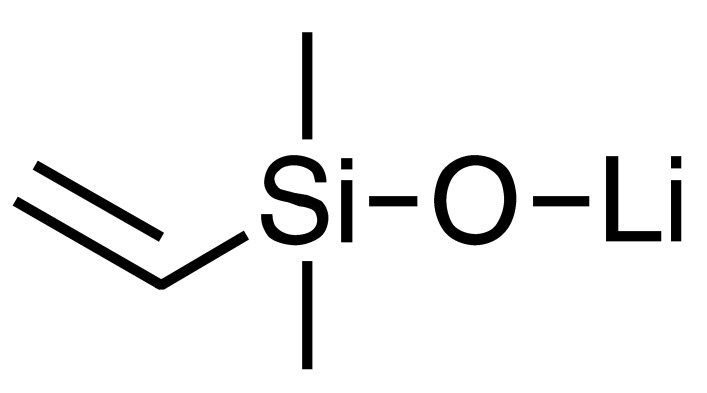		Goff [73] Okawa [73,74] Arkles [62]
Vinylbutylmethylsilanol lithium salt	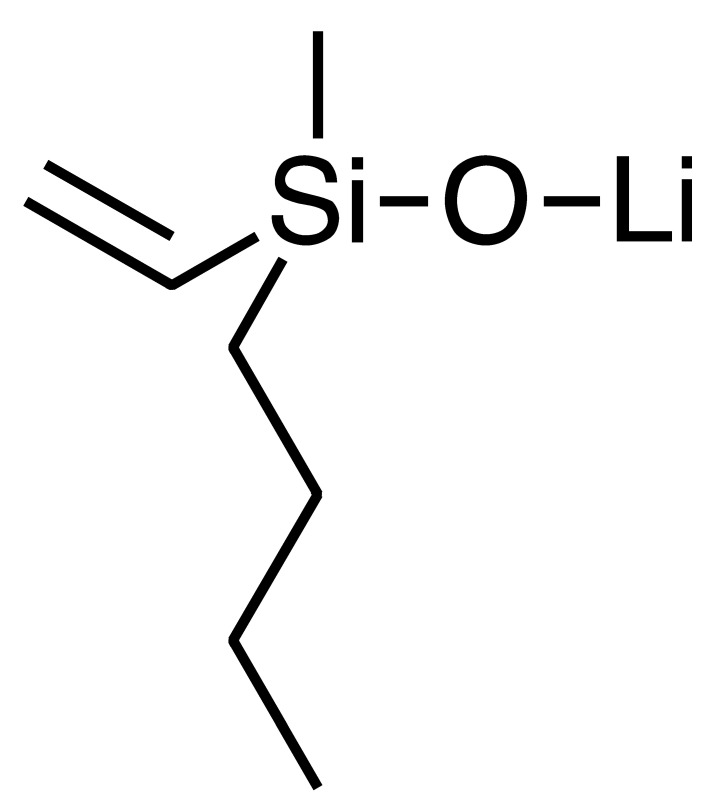		Arkles [62]
Hydridobutylmethylsilanol lithium salt	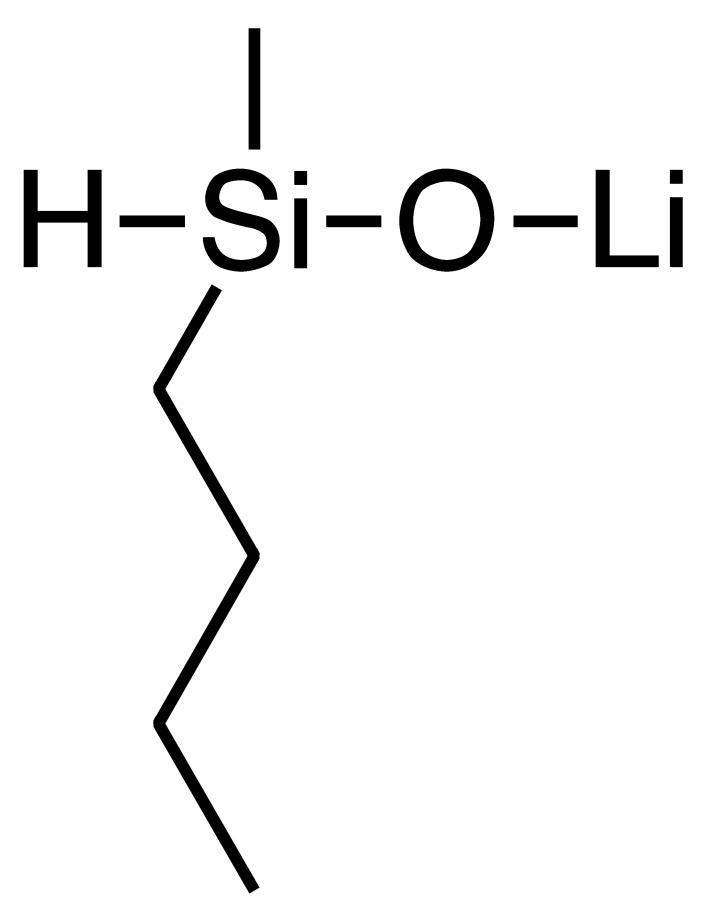		Arkles [62]
1-Hexenyl, 4-Hydroxyl-octamethyltetrasiloxane lithium salt	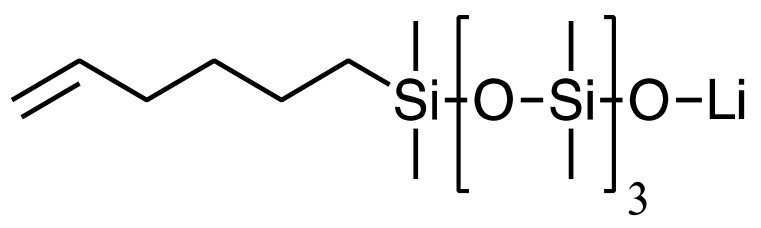		Okawa [74]
**Other**			
Lithium anion of Na pentanedionate	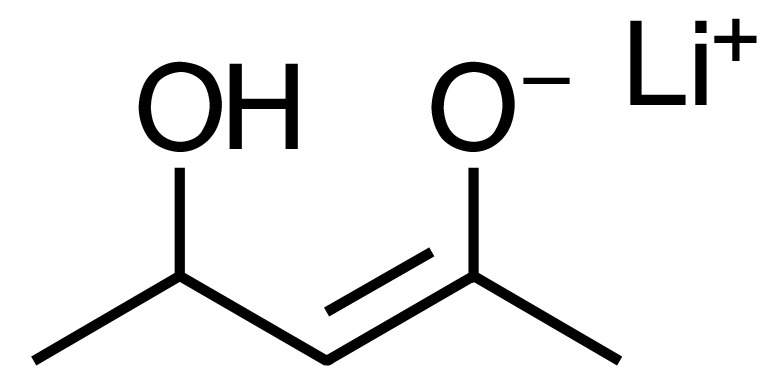	Forms Na AcAc-terminated polymer, but not stable after neutralization	Kumar [75]
1,5,7-Triazabicyclo [4.4.0] dec-5-ene	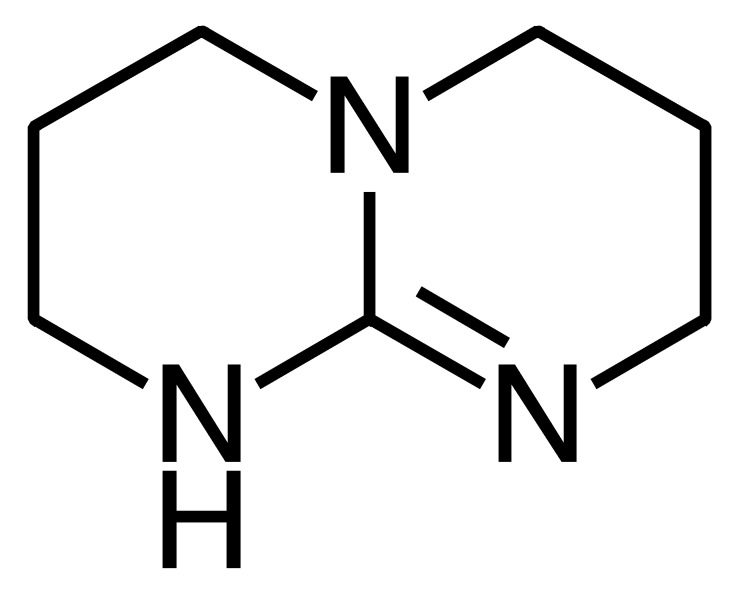	Used in the polymerization of monodisperse silanol-terminated siloxanes	Lohmeijer [14]
1,3-Trimethylene-n-propylguanidine	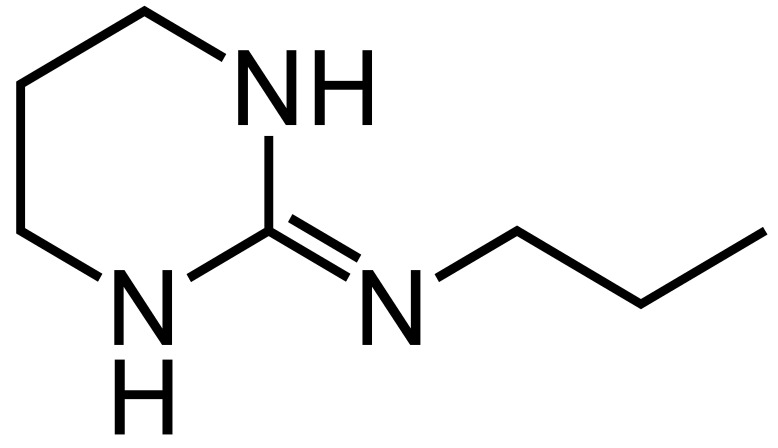	Used in combination with water and silanols	Fuchise [15,76]

**Table 3:** Commonly Reported Macromers.

Asymmetric Monofunctional Siloxanes						
Code *	Description	Molecular Weight, Mn	Viscosity, cSt	Density	Refractive Index	Reference
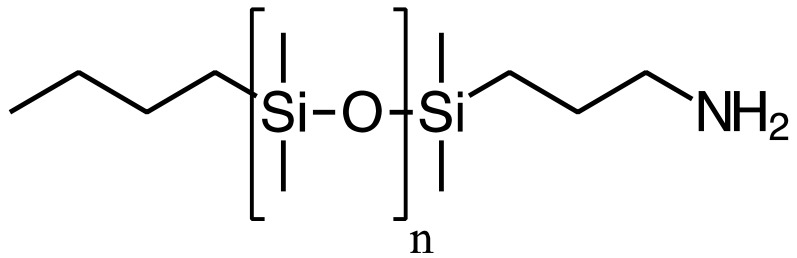						
MCR-A11	monoAMINOPROPYL TERMINATED POLYDIMETHYLSILOXANE	800–1000	8–12	0.82	1.411	[79,80,81]
MCR-A12	monoAMINOPROPYL TERMINATED POLYDIMETHYLSILOXANE	2000	18–25	0.97	1.411	[79,81]
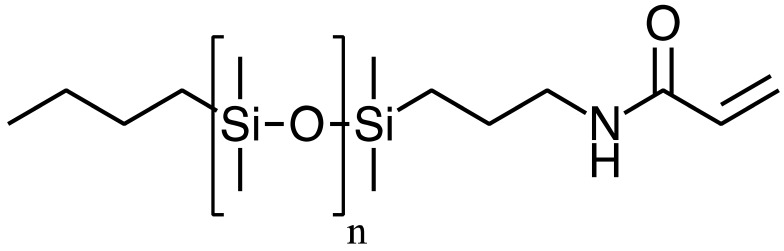						
MCR-W15	monoACRYLAMIDOPROPYL TERMINATED POLYDIMTHYLSILOXANE	1000–1500	50–75	0.96	1.418	[79]
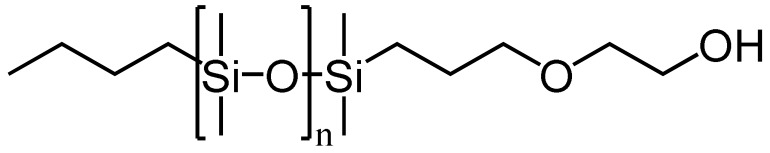						
MCR-C12	monoCARBINOL TERMINATED POLYDIMETHYLSILOXANE	1000	15–20	0.96	1.409	[79,82]
MCR-C18	monoCARBINOL TERMINATED POLYDIMETHYLSILOXANE	5000	80–90	0.97	1.405	[79,82,83,84]
MCR-C22	monoCARBINOL TERMINATED POLYDIMETHYLSILOXANE	10,000	250	0.98	1.404	[79,81,82,85]
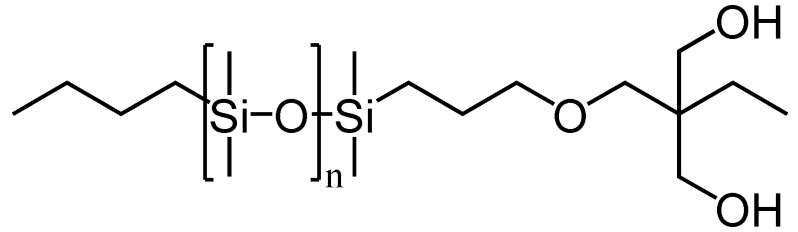						
MCR-C61	monoDICARBINOL TERMINATED POLYDIMETHYLSILOXANE	1000	50–60	0.97	1.417	[79,86]
MCR-C62	monoDICARBINOL TERMINATED POLYDIMETHYLSILOXANE	5000	100–125	0.97	1.409	[79,86,87]
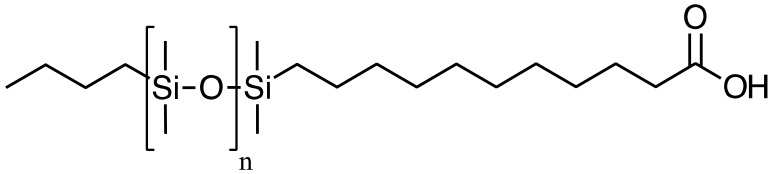						
MCR-B12	monoCARBOXYDECYL TERMINATED POLYDIMETHYLSILOXANE	1500	20	0.94	1.415	[79,88]
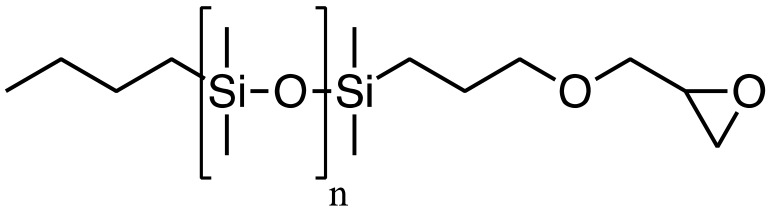						
MCR-E11	Mono(2,3-EPOXY)PROPYLETHER TERMINATED POLYDIMETHYLSILOXANE	1000	10–15	0.96	1.410	[79,89,90]
MCR-E21	Mono(2,3-EPOXY)PROPYLETHER TERMINATED POLYDIMETHYLSILOXANE	5000	120	0.97	1.408	[79]
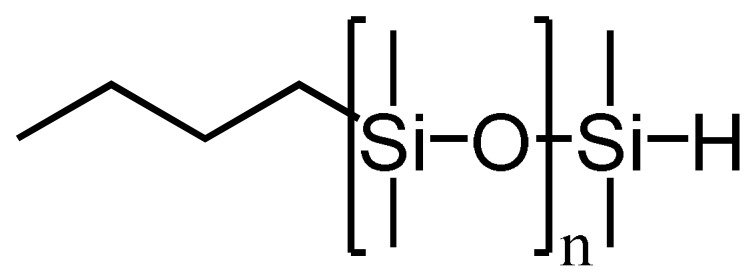						
MCR-H07	MonoHYDRIDE TERMINATED POLYDIMETHYLSILOXANE	800–900	5–8	0.96	1.404	[60,79]
MCR-H11	MonoHYDRIDE TERMINATED POLYDIMETHYLSILOXANE	900–1100	8–12	0.96	1.407	[60,79,91]
MCR-H21	MonoHYDRIDE TERMINATED POLYDIMETHYLSILOXANE	4500–5000	80–120	0.96	1.411	[79]
MCR-H22	MonoHYDRIDE TERMINATED POLYDIMETHYLSILOXANE	10,000	160–220	0.98	1.411	[79]
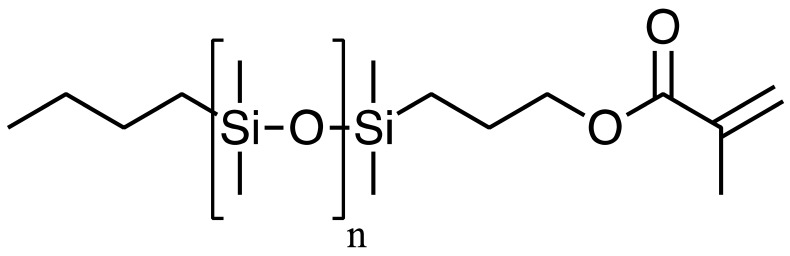						
MCR-M07	MonoMETHACRYLOXYPROPYL TERMINATED POLYDIMETHYLSILOXANE	600–800	6–9	0.96	1.416	[60,79,92,93,94,95,96,97]
MCR-M11	MonoMETHACRYLOXYPROPYL TERMINATED POLYDIMETHYLSILOXANE	800–1000	10–11	0.96	1.411	[60,70,79,92,93,95,96,97,98,99,100,101,102,103,104,105,106,107,108]
MCR-M17	MonoMETHACRYLOXYPROPYL TERMINATED POLYDIMETHYLSILOXANE	5000	70–80	0.97	1.406	[54,70,79,89,90,93,95,96,97,108,109,110]
MCR-M22	MonoMETHACRYLOXYPROPYL TERMINATED POLYDIMETHYLSILOXANE	10,000	150–200	0.97	1.405	[54,79,93,94,96,97]
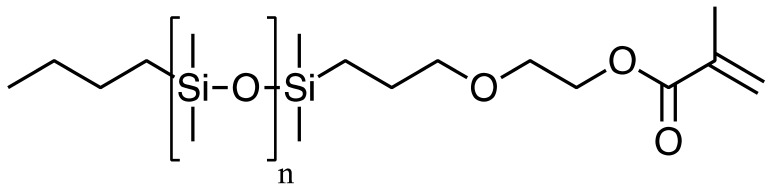						
MCR-MXe11	MonoMETHACRYLOXYETHOXY-PROPYL TERMINATED POLYDIMETHYLSILOXANE	900–1000	9–10	0.95	1.415	None?
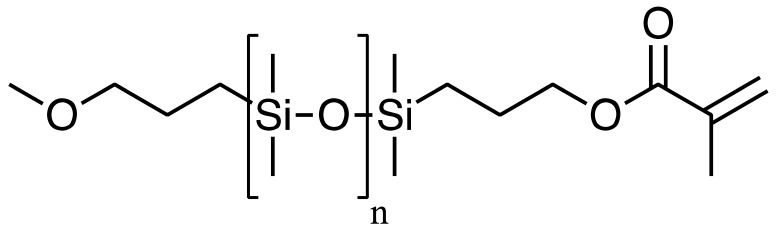						
MCR-MXt11	MonoMETHACRYLOXYPROPYL, MonoMETHOXYPROPYL TERMINATED POLYDIMETHYLSILOXANE	800–1000	8–12	0.96	1.430	[60,70]
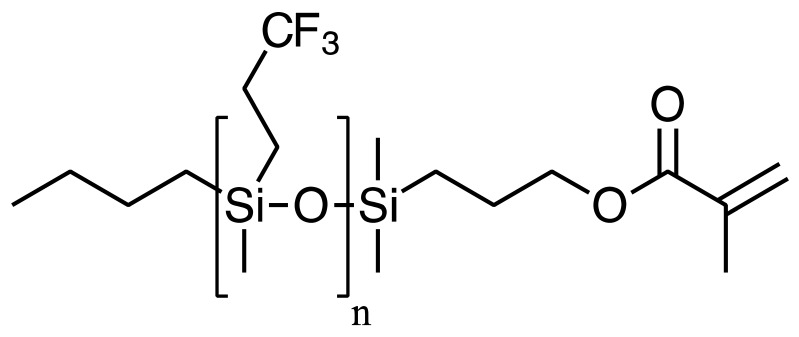						
MFR-M15	MonoMETHACRYLOXYPROPYL TERMINATED POLYTRIFLUOROPROPYLMETHYLSILOXANE	800–1000	50–70	1.09	1.398	[59,60,70,79,93,100]
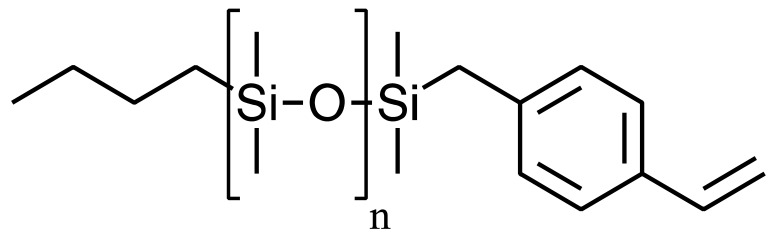						
MCR-ST11	MonoSTYRYL TERMINATED POLYDIMETHYLSILOXANE	800–1000	8–15	0.95	1.429	[99,102]
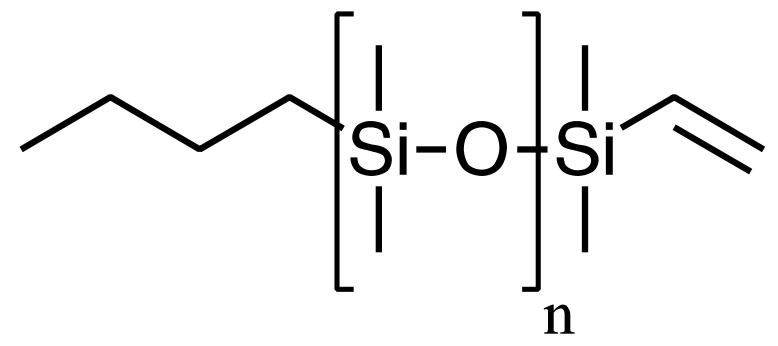						
MCR-V21	MonoVINYL TERMINATED POLYDIMETHYLSILOXANE	5500–6500	80–120	0.97	1.403	[79,91,111]
MCR-V25	MonoVINYL TERMINATED POLYDIMETHYLSILOXANE	15,000–20,000	400–600	0.97	1.403	[79]
MCR-V41	MonoVINYL TERMINATED POLYDIMETHYLSILOXANE	55,000–65,000	8000–12,000	0.98	1.404	[79]
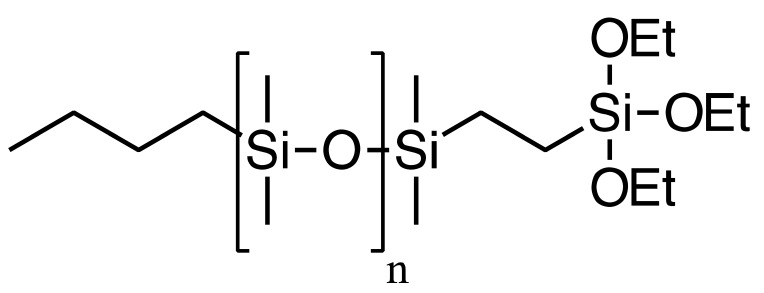						
MCR-XT11	MonoTRIETHOXYSILYLETHYL TERMINATED POLYDIMETHYLSILOXANE	500–1000	16–24	0.97	1.412	[79]
**Symmetric Monofunctional Siloxanes**						
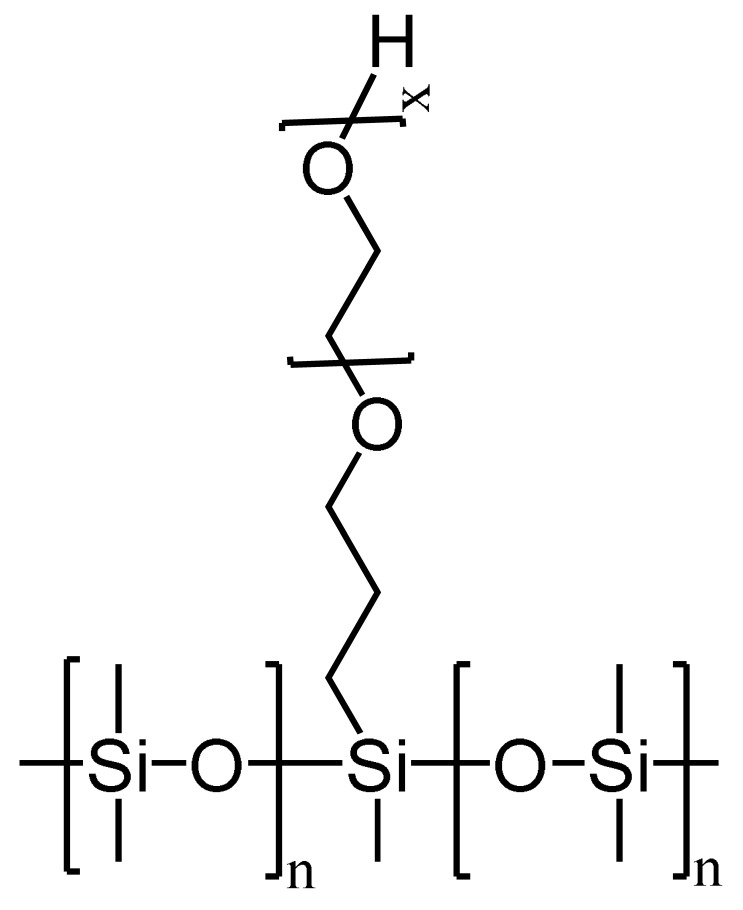						
MCS-C13	MonoCARBINOL FUNCTIONAL POLYDIMETHYLSILOXANE, symmetric	550–650	35–40	1.02	1.446	[79]
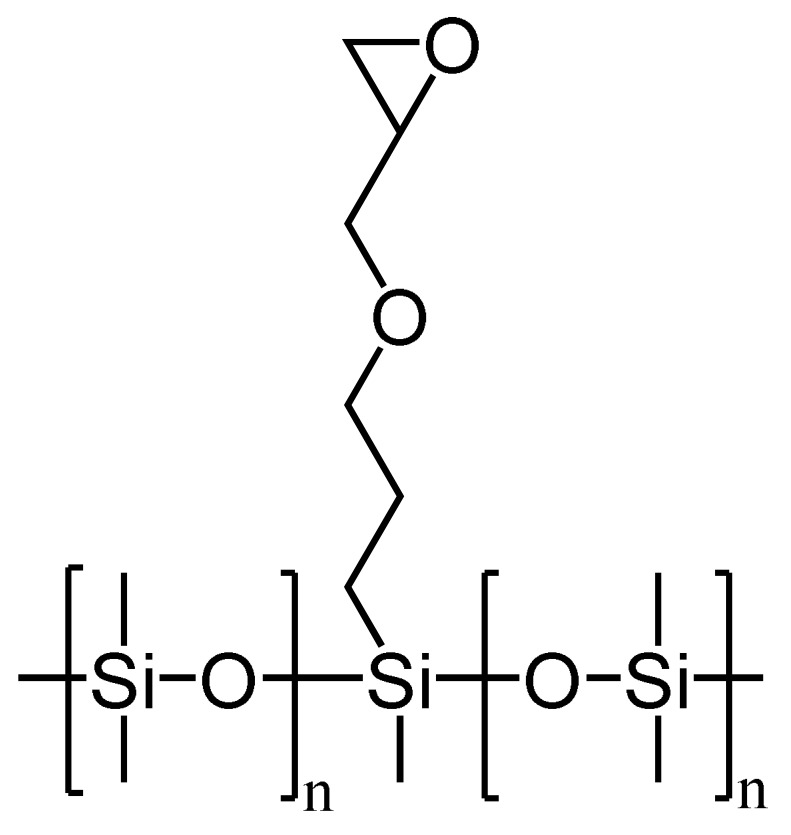						
MCS-E15	Mono(2,3-EPOXY)PROPYL ETHER FUNCTIONAL POLYDIMETHYLSILOXANE, symmetric	800–900	45–55	1.09	1.398	[59,70,79]
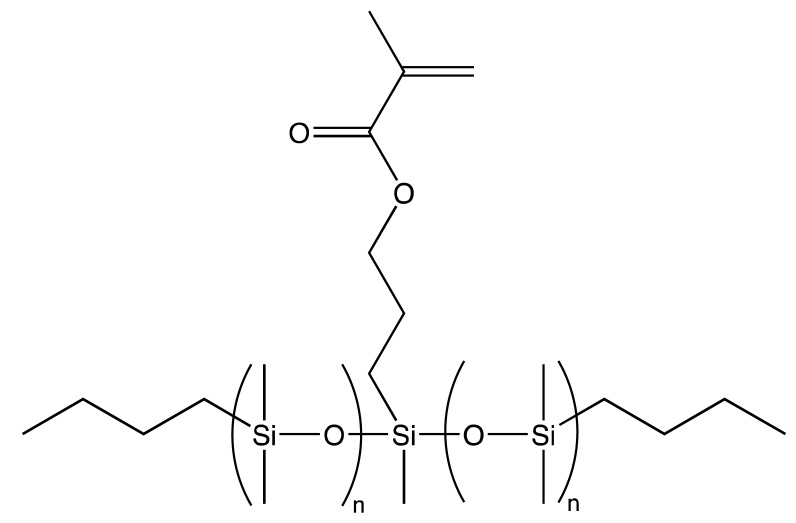						
MCS-M11	MonoMETHACRYLOXYPROPYL FUNCTIONAL POLYDIMETHYLSILOXANE, symmetric	800–1000	7–9	0.93	1.417	[59,60,70,79,93]
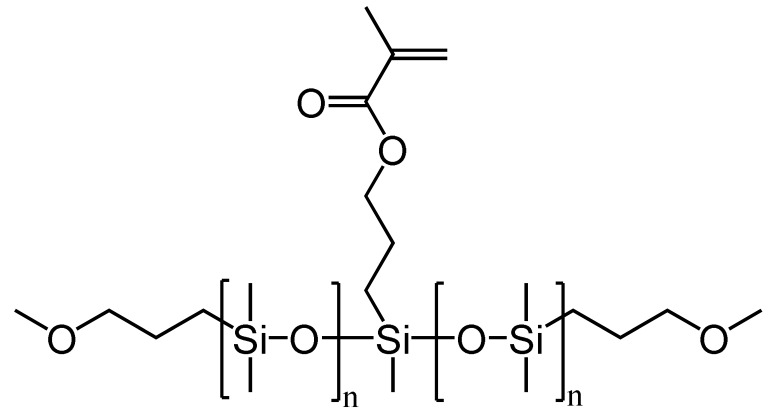						
MCS-MXt11	MonoMETHACRYLOXYPROPYL FUNCTIONAL POLYDIMETHYLSILOXANE, METHOXYPROPYL TERMINATED, symmetric	900–1100	8–12	0.96	1.43	[60,79]
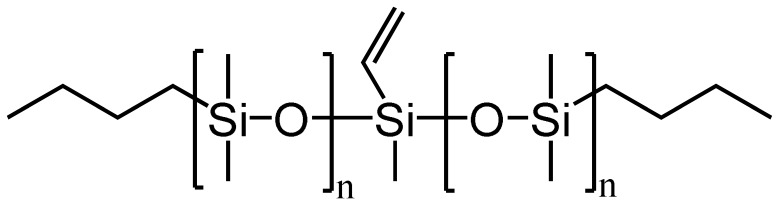						
MCS-V212	MonoVINYL TERMINATED POLYDIMETHYLSILOXANE, symmetric	1200–1400	16–24	0.97	1.419	[79]
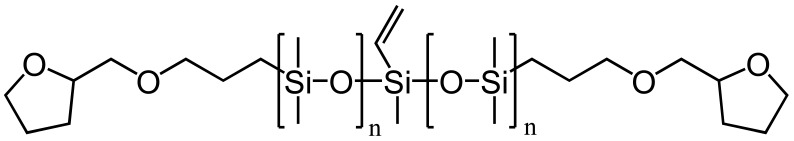						
MCS-VF14	monoVINYL FUNCTIONAL POLYDIMETHYLSILOXANE, TETRAHYDROFURFURYLOXYPROPYL TERMINATED—symmetric	1000–1200	30–40	0.976	1.4141	[61,79,87,112]
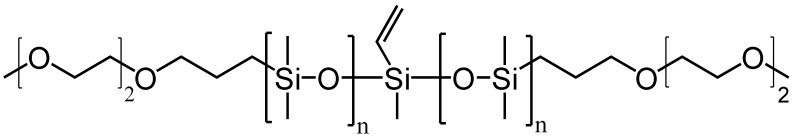						
MCS-VXt15	MonoVINYL FUNCTIONAL POLYDIMETHYLSILOXANE, METHOXY(DIETHYLENEOXIDE)PROPYL TERMINATED, symmetric	1000–1200	40–60	0.96	1.42	[61,79,87,112]

* In order to provide a concise description of the macromer systems discussed, the following designation system was adopted: the first two letters, MC, indicate macromer M structure with a C conventional dimethylsiloxane repeat unit; the third letter indicates whether it is asymmetric (R), symmetric (S), or T-structure (T). Macromer functionality and viscosity are indicated after a hyphen, as follows: (1) letters indicate functionality, where M is methacrylate, H is hydride, C is carbinol, V is vinyl, and E is methoxy; (2) numbers are the viscosity ranges of the macromer in powers of 10, followed by the first significant figure. For example, MCR-H25 indicates a macromer, an asymmetric architecture with hydride functionality, and a viscosity of 10^2^ × 5 or 500 cSt. These designations provide a facile method of discussing comparative structures but are not intended to be detailed structural descriptions of the polymers. To remove ambiguity for ethyleneoxy designated X spacer groups, the lower-case letter t, for “tipped”, or e, for “embedded”, is used.

**Table 4:** Monodisperse Difunctional Macromers.

Heterobifunctional Siloxanes						
Code	Description	Molecular Weight, Mn	Viscosity, cSt	Density	Refractive Index	Reference
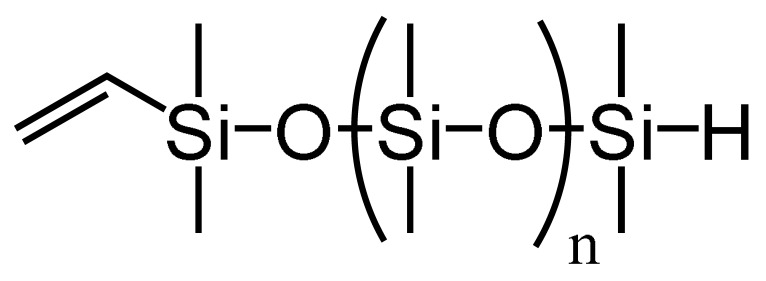						
DMS-HV15	monoVINYL-monoHYDRIDE terminated POLYDIMETHYLSILOXANE	2000–3000	40–60	0.96	1.404	[62,73,79,113,114]
DMS-HV22	monoVINYL-monoHYDRIDE terminated POLYDIMETHYLSILOXANE	10,000	150–250	0.97	1.403	[62,73,79,113,114,115,116]
DMS-HV31	monoVINYL-monoHYDRIDE terminated POLYDIMETHYLSILOXANE	25,000	600–1000	0.97	1.403	[62,73,79,114]
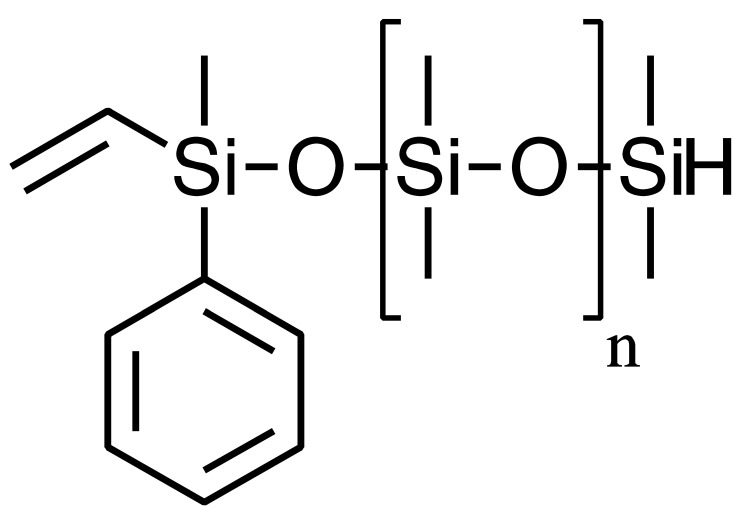						
PMM-HV12	α-monoVINYL,monoPHENYL-ω-monoHYDRIDE terminated POLYDIMETHYLSILOXANE	2000	20	0.97	1.414	[62,79]

**Table 5:** Reactivity Ratios of Methacrylate Functional Siloxane Macromers.

Siloxane Macromer	Comonomer	Reactivity Ratio (r1:r2) *
MCR-M11	Methylmethacrylate	nm **:1.60
MCR-M22		nm **:2.10
MCR-M11	Styrene	0.26:1.07
MCR-M11	Acrylonitrile	5.40:0.89

* Rate constants—M1M1/M1M2: M2M2/M2M1; ** no meaningful results.

**Table 6:** Solubility of Methacrylate Functional Silicone Macromers *.

Siloxane Macromer—Methacrylate Functional	Macromer Solubility in Hydrogel Monomers		Water Solubility in Macromer (ppm)
	Dimethylacrylamide (DMA)	Hydroxyethylmethacrylate (HEMA)	
MCR-M11 (asymmetric)	4%	1%	2000
MCS-M11 (symmetric)	8%	2%	3500
MFR-M15 (fluorinated asymmetric)	100% (miscible)	2%	9000
MFS-M15 (fluorinated symmetric)	100% (miscible)	3%	10,000
MCS-MXt11 (methoxy-tipped, symmetric)	100% (miscible)	100% (miscible)	10,000

* Author’s work, previously unreported.

**Table 7:** Silicone Hydrogel Contact Lens Technology.

Technology Generation	Proprietary Name	Material	Manufacturer	Launch Date	Water Content (%)	Dk	O_2_ Transport Enhancer	Hydrophilic Component *	Refs.
1	PureVision	Balafilcon A	Bausch & Lomb	1998	36	91	TRIS, Difunctional siloxane	NVP amino acid monomer	
	Focus Night & Day	Lotrfilcon A	CIBA Vision	1999	24	140	TRIS, Difunctional fluorosiloxane	DMA	Nicolson [124]
	AirOptix	Lotrafilcon B	CIBA Vision	1999	33	110	TRIS, Difunctional fluorosiloxane	DMA	Nicolson [124]
2	Biofinity	Cofilcon A	Cooper vision	2007	48	128	Monofunctional siloxane macromer	VMA	Ueyama [125] Iwata [126]
	Acuvue OASYS	Senfilcon A	JJVC	2004	38	103	SiGMA monomer, monofunctional siloxane macromer (MCR-M11)	DMA, HEMA, PVP	Vanderlaan [101,123]
	PremuiO	Amofilcon A	Menicon	2007	40	129	Difunctional fluorosiloxane macromer	DMA	
	Acuvue Advance	Gayfilcon A	JJVC	2004	47	60	SiGMA monomer, monofunctional siloxane macromer (MCR-M11)	DMA, HEMA, PVP	Vanderlaan [101,123]
3	1-DAY Acuvue TruEYE	Narfilcon A	JJVC	2008	46	100	SiGMA monomer, monofunctional siloxane macromers (OH-mPDMS, MCR-M11)	DMA, HEMA, PVP	Vanderlaan [101,123]
	Clariti 1-DAY	Somofilcon	Cooper vision	2014	56	60	Monofunctional siloxane macromer, difunctional siloxane	HEMA	Broad [100]
	Avaira	Enfilcon	Cooper vision	2011	46	100	Difunctional fluorosiloxane	VMA	
	Ultra	Samfilcon A	Bausch & Lomb	2014	46	114	TRIS, Difunctional siloxane, monofunctional carbosiloxane macromer	Unknown	Awasti [55,127]
	DAILIES TOTAL1	Delfilcon A	Alcon	2013	33	140	Unknown	DMPC, DMA	
	MyDay	Stenfilcon A	Cooper vision	2013	54	80	Difunctional PEG-PDMS copolymer, monofunctional siloxane macromer	VMA	
	Acuvue Vita	Senfilcon C	JJVC	2016	41	103	SiGMA monomer, monofunctional siloxane macromer (MCR-M11)	DMA, HEMA, PVP	

* DMA—Dimethylacrylamide; DMPC—(1,2-dimyristoyl-sn-glycero-3-phosphocholine; HEMA—Hydroxyethylmethacrylate; NVP—N-Vinylpyrrolidone; PVP—Polyvinylpyrrolidone; SiGMA—3-(3-Methacryloxy-2-hydroxypropoxy)propylbis(trimethylsiloxy)methylsilane; TRIS—Methacryloxypropyltris(trimethylsiloxy)silane; VMA—N-Vinylmethylacetamide.

**Table 8:** Comparing Contact Angle, Abrasion, and Friction in an IPDI-based Urethane.

	Control	A	B	C
Description		Blend	Block Polymer	Pendant/Side-Chain
Silicone Component		Silicone Fluid	Telechelic Carbinol	Dicarbinol Terminated Asymmetric Macromer
Abrasion Test Failure, cycles	<100	<100	<100	4200
Coefficient of Friction	0.61	0.12	0.74	0.16
Contact Angle, water before abrasion	56°	83°	72°	82°
Contact Angle, water after abrasion	55°	55°	72°	80°

Control (no siloxane); (A) introduction of 50 cSt dimethylsiloxane fluid as a non-reactive additive; (B) telechelic siloxane MW 5000 (DMS-C21) added to form a soft-block copolymer; (C) macromer MW 5000 (MCR-C62) forming a brush polymer with siloxane pendant.

## Data Availability

Not applicable.
